# Chromosomal copy number heterogeneity predicts survival rates across cancers

**DOI:** 10.1038/s41467-021-23384-6

**Published:** 2021-05-27

**Authors:** Erik van Dijk, Tom van den Bosch, Kristiaan J. Lenos, Khalid El Makrini, Lisanne E. Nijman, Hendrik F. B. van Essen, Nico Lansu, Michiel Boekhout, Joris H. Hageman, Rebecca C. Fitzgerald, Cornelis J. A. Punt, Jurriaan B. Tuynman, Hugo J. G. Snippert, Geert J. P. L. Kops, Jan Paul Medema, Bauke Ylstra, Louis Vermeulen, Daniël M. Miedema

**Affiliations:** 1 Department of Pathology, Cancer Center Amsterdam, Amsterdam UMC, Vrije Universiteit Amsterdam, Amsterdam, The Netherlands; 2 LEXOR, Center for Experimental and Molecular Medicine, Cancer Center Amsterdam and Amsterdam Gastroenterology & Metabolism, Amsterdam UMC, University of Amsterdam, Amsterdam, The Netherlands; 3 Oncode Institute, Amsterdam, The Netherlands; 4 Hubrecht institute—KNAW and University Medical Center Utrecht, Utrecht, The Netherlands; 5 Center for Molecular Medicine, University Medical Centre Utrecht, Utrecht, The Netherlands; 6 MRC Cancer Unit, University of Cambridge, Cambridge, UK; 7 Department of Epidemiology, Julius Center for Health Sciences and Primary Care, University Medical Center Utrecht, Utrecht, The Netherlands; 8 Department of Surgery, Amsterdam UMC, Vrije Universiteit Amsterdam, Amsterdam, The Netherlands

**Keywords:** Cancer genomics, Tumour heterogeneity, Computational biology and bioinformatics

## Abstract

Survival rates of cancer patients vary widely within and between malignancies. While genetic aberrations are at the root of all cancers, individual genomic features cannot explain these distinct disease outcomes. In contrast, intra-tumour heterogeneity (ITH) has the potential to elucidate pan-cancer survival rates and the biology that drives cancer prognosis. Unfortunately, a comprehensive and effective framework to measure ITH across cancers is missing. Here, we introduce a scalable measure of chromosomal copy number heterogeneity (CNH) that predicts patient survival across cancers. We show that the level of ITH can be derived from a single-sample copy number profile. Using gene-expression data and live cell imaging we demonstrate that ongoing chromosomal instability underlies the observed heterogeneity. Analysing 11,534 primary cancer samples from 37 different malignancies, we find that copy number heterogeneity can be accurately deduced and predicts cancer survival across tissues of origin and stages of disease. Our results provide a unifying molecular explanation for the different survival rates observed between cancer types.

## Abstract (web summary)

Intratumour heterogeneity (ITH) is associated with worse prognosis in cancer, and efficient frameworks to measure it are needed. Here the authors develop a method to estimate copy number heterogeneity, and propose that it is driven by chromosomal instability and can predict pan-cancer survival.

## Introduction

The abundance and diversity of genomic aberrations in cancers is enormous^1^. Identifying common characteristics across cancer genomes that define patient survival therefore remains challenging. In particular, malignancies from different cancer types are molecularly highly distinct; therefore, most biomarkers are restricted to a single type of cancer^2^. The hallmark process of genomic instability and its direct consequence ITH are generic features of cancers that might associate with poor prognosis, and could stratify patients and inform on tumour biology in a pan-cancer setting^3–6^.

Genomic instability and intra-tumour heterogeneity (ITH) occur at different levels, ranging from single-nucleotide variations (SNVs) to chromosomal losses and duplications. Most studies of ITH have focused on the single-nucleotide scale, and some reported a relation to survival^7–12^. However, the reliability of these methods based on mutation frequency calling is under debate^13–15^. In cancers from various organs, it has been shown that chromosomal copy number variations (CNVs) are heterogeneous within tumours^16–19^, and might be more important for patient outcome^18,20^. However, a robust and comprehensive method to infer ITH from a single copy number measurement is currently lacking, limiting the possibility to study large numbers of patients in a pan-cancer setting.

Here, we introduce an approach to quantify ITH from a single copy number profile. This method allowed us to uncover that ITH at the copy number level results from ongoing chromosomal instability and underlies cancer prognosis in individual cancer types as well as across distinct malignancies.

## Results

### Accurate ITH measurement from a single copy number profile: CNH

**Fig. 1:**
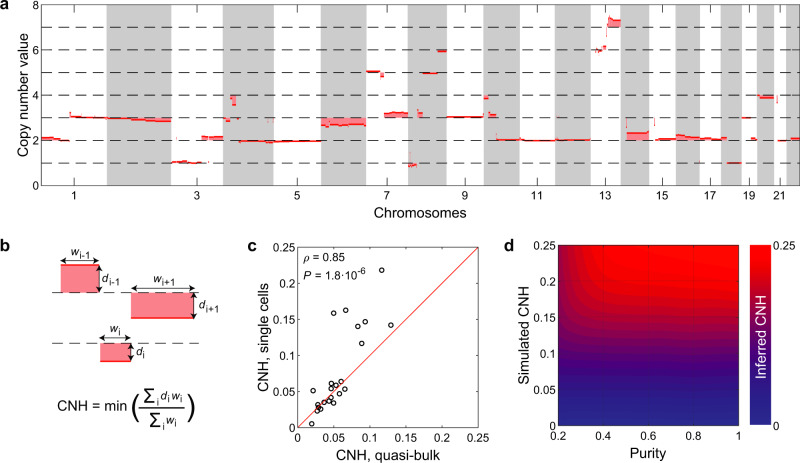
Intra-tumour heterogeneity measurement from single copy number profile. **a** Example of an absolute copy number profile. Deviations of absolute copy numbers from integer values are indicated by the shaded areas and reflect heterogeneity. **b** Scheme showing the formal definition of copy number heterogeneity (CNH). The minimum is taken over purity and ploidy. *w*, segment width. *d*, distance of segment to the closest integer. **c** CNH obtained from the pooled reads of single cells (quasi-bulk) correlates well to CNH determined from direct comparison of karyotypes of individual cells. Spearman’s rank correlation is reported. Red line is the diagonal, ‘CNH, single cell’ = ‘CNH, quasi-bulk’. **d** CNH of simulated copy number profiles can be accurately inferred, independent of tumour purity. Source data are provided as a Source Data file.

To derive ITH from a single tissue sample copy number profile, we postulate that each individual cell has strictly integer chromosomal copy numbers (i.e. 0, 1, 2,…). Hence, completely homogeneous samples also have integer copy number values, and deviations from integer values in an absolute copy number profile reflect heterogeneity in the malignant cell population (Fig. 1a). To quantify copy number heterogeneity (CNH) within malignancies, we infer tumour ploidy, sample purity and absolute copy numbers from a normalized and segmented copy number profile^21–23^. For a range of ploidies (1.5, 1.55,.., 5) and malignant cell purities (0.2, 0.21,.., 1), we calculate the absolute copy number profile, measure the distance of each segment to the closest integer and determine the average distance weighted by segment lengths (see ‘Methods’ for details). CNH is then defined as the minimum of the average weighted distances taken over all ploidies and purities (Fig. 1b).

CNH can be interpreted as the average fraction of malignant cells that differ by one copy from the mode copy number value at each position of the genome. It integrates the fraction of heterogeneous malignant cells and the fraction of the genome that is heterogeneous in one quantitative score. We verify with single-cell karyotype sequencing, multi-region copy number data and simulated copy number profiles that CNH is accurately determined by our inference procedure (Fig. 1c and Supplementary Fig. 1a–c)^18,24^. Non-malignant cells in a cancer sample can affect estimates of ITH^14^. We expand our simulations by including increasing fractions of non-malignant cells and find that CNH is robust to varying purities (malignant cell fraction between 0.2 and 1, Fig. 1d). Next, we assess the independence of tumour purity in a dataset containing 253 samples from patients with ovarian cancer, where tumour purity was determined separately from the copy number data^25^. Inference of CNH using purity as a free parameter (our standard procedure), or fixing purity around the reported value for each sample gives highly concordant results (Supplementary Fig. 1d, e). More generally, multiple combinations of ploidy and purity can result in good fits of absolute copy number profiles, i.e. with small segment distances to integer values (Supplementary Fig. 1f, g). Importantly, CNH is by definition essentially identical for the different solutions (Supplementary Fig. 1h, i). We further note that CNH can be inferred from allele-specific as well as total copy numbers (default), and that filtering of noisy segments can be implemented before the inference of CNH to improve accuracy (Supplementary Figs. 2a–h). Finally, we show that CNH can be accurately determined from stored FFPE material, independent of the measurement platform used to obtain copy number data (Supplementary Fig. 2i). A copy number profile derived from a single sample with unknown cancer cell purity is thus sufficient to accurately determine CNH.

### Ongoing chromosomal instability underlies CNH

**Fig. 2:**
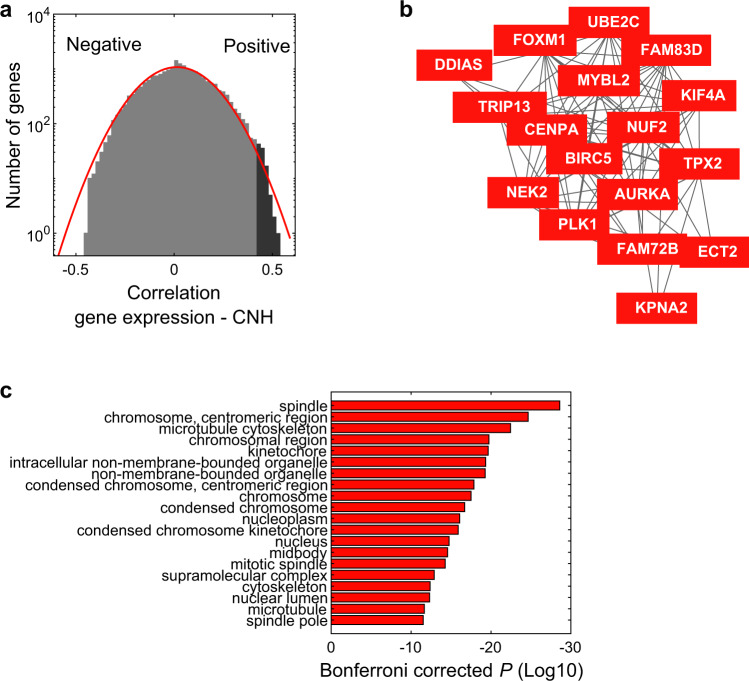
Chromosomal instability underlies CNH. **a** Histogram showing Spearman’s rank correlations of gene expressions to copy number heterogeneity (CNH). The correlation is calculated for all genes, on 8968 primary cancer samples in TCGA, which have combined copy number and gene-expression data. The red line is a Gaussian fit to the distribution. **b** Co-functionality analysis reveals a cluster of genes positively correlated with CNH. **c** Most significant cellular component gene ontologies of the genes most positively correlated with CNH (*ρ* > 0.42) indicated by the dark-grey colour in **a**. Source data are provided as a Source Data file.

Does heterogeneity in copy numbers result from ongoing chromosomal instability, or from the coexistence of multiple clones that emerged at some point during cancer evolution^9,24,26,27^? Gene-expression profiles can be used as a proxy of chromosomal instability in malignancies^28^. We explore the relation between chromosomal instability and CNH in an unbiased way using gene expression and copy number data from The Cancer Genome Atlas (TCGA) (8968 patients with copy number and gene-expression data). We determine CNH from the copy number profiles of all primary cancers and correlate CNH to the expression level of all genes (Supplementary Data 1, 2). The correlation of gene-expression levels is close to normally distributed, but the slight enrichment of positive correlations suggests a group of genes related to CNH (Fig. 2a). Indeed, the top genes positively correlated with CNH form a functionally related cluster, including *AURKA*, a gene encoding a kinase that is targeted to mitotic spindle microtubules by TPX2 in a highly conserved way (Fig. 2b)^29–31^. Gene ontology analysis of the tail of the distribution (genes with Spearman’s rank correlation >0.42) reveals that high CNH is characterized by chromosomal instability (Fig. 2c and Supplementary Fig. 3a–c)^28,32,33^. In contrast, no particular ontology is significantly associated with the most negatively correlated genes. This gene-expression analysis suggests that chromosomal instability drives CNH in a malignancy. Using live imaging of cell divisions in organoids, we find that indeed missegregation of chromosomes is associated with CNH (Supplementary Fig. 3d). While our results indicate that chromosomal instability underlies CNH, we note that other factors also contribute to CNH. For example, we find that haematological malignancies show evidence for high chromosomal instability and low CNH, relative to other cancer types (Supplementary Fig. 3e). The competition between malignant cells in haematological malignancies is less spatially constrained, which facilitates selective sweeps that reduce heterogeneity and could explain the low values of CNH observed in these malignancies^34^.

**Fig. 3:**
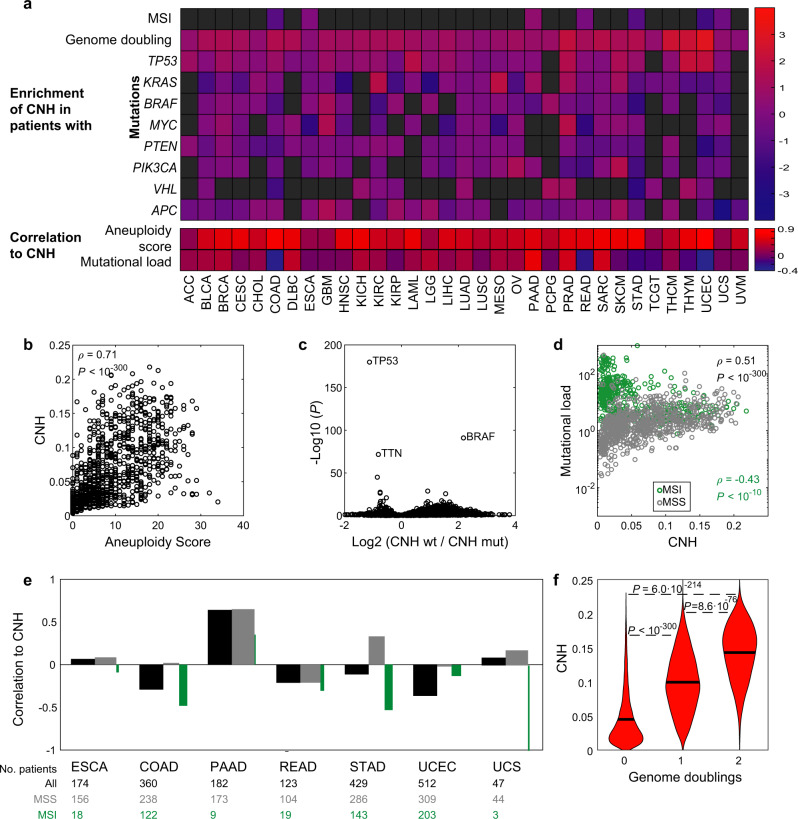
Genetic characterization of CNH. **a** Genomic associations per type for all 33 cancer types in TCGA. Upper panel: enrichment of copy number heterogeneity (CNH) in microsatellite-instable (MSI), genome-doubled or mutated cancers versus non-MSI, non-genome-doubled and non-mutated cancers, respectively. The enrichment of CNH is defined as the log2 of the ratio between group medians with and without event. Lower panel: Spearman’s rank correlation of CNH to aneuploidy and mutational load per cancer type. **b** Pan-cancer Spearman’s rank correlation between CNH and the aneuploidy score. **c** Identification of mutated genes related to CNH in a pan-cancer setting. Malignancies are grouped as wild type or mutated for each gene. The relative difference in median CNH of these groups (horizontal axis) and the corresponding significance calculated by the Wilcoxon rank-sum test (vertical axis) are shown. **d** Spearman’s rank correlation between CNH and mutational load in MSI tumours (green) and non-MSI cancers (grey). **e** Spearman’s rank correlation between CNH and mutational load per cancer type for all tumours (black), non-MSI tumours (grey) and MSI tumours (green). The relative width of the grey and green bars reflects the ratio of MSI/non-MSI tumours in each type. **f** Malignancies that have undergone genome doubling have a higher CNH. Groups are compared by the Wilcoxon rank-sum test. Source data are provided as a Source Data file.

Next, we characterize the relation between CNH and various genomic aberrations. As expected, heterogeneity in copy numbers requires CNVs (Fig. 3a, b)^35^. Furthermore, we identify mutations in *TP53* as most significantly associated with high CNH, suggesting that the ability of a cell to cope with aneuploidy is also vital to CNH (*P* < 10^−150^, Fig. 3a, c). *TP53* is the most frequently mutated gene across cancers and the safeguard of genome stability, protecting against both CNVs and SNVs^35^. Indeed, we find that CNH positively correlates with mutational load, except for microsatellite-instable (MSI) tumours in which the correlation is negative (Fig. 3a, d, e). Genome doubling can enhance chromosomal instability^36^. We find higher CNH for genome-doubled malignancies, which further underlines the relation between CNH and chromosomal instability (Fig. 3a, f).

### CNH is a unifying predictor of survival for cancer patients

**Fig. 4:**
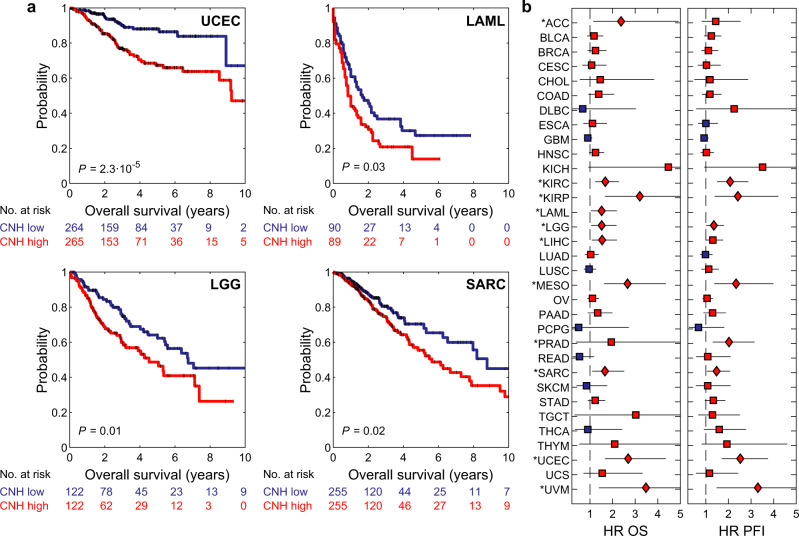
CNH is prognostic for survival in most types of cancer. **a** Kaplan–Meier plots of overall survival for uterine corpus endometrial cancer (UCEC), sarcoma (SARC), low-grade glioma (LGG) and acute myeloid leukaemia (LAML). The homogeneous (blue lines) and heterogeneous (red lines) groups were compared by the two-sided log-rank test. **b** Hazard ratios (HR) of copy number heterogeneity (CNH) for overall survival (OS, left panel) and progression-free interval (PFI, right panel) of all 33 cancer types in TCGA. Hazard ratios were calculated using univariate Cox proportional-hazard models. Red (blue) symbol indicates that the CNH-high (low) group has poorer survival. Significance is indicated by diamonds and marked with an asterisk per type. Error bars represent 95% confidence intervals of hazard ratios as given by the two-sided Wald test. Patients are split in two groups of equal size based on rank-ordered CNH in these survival analyses. Source data are provided as a Source Data file.

Genomic heterogeneity in the malignant cell population is a source for cancer evolution, regardless of the tissue of origin. As cancer evolution impairs patient survival and treatment efficacy, we asked if our CNH measure associates with survival for the different cancer types documented in TCGA^37^. For each sample, we calculate the CNH and rank-order patients accordingly. We split patients into two groups of equal size for each type of malignancy. We find that CNH distinguishes patients with poor and good prognosis in highly different cancer types, such as uterine corpus endometrial carcinoma (UCEC), acute myeloid leukaemia (LAML), sarcoma (SARC) and brain lower-grade glioma (LGG) (Fig. 4a). For the large majority of cancer types, CNH is associated with poor prognosis for progression-free interval (PFI) and overall survival (OS) (Fig. 4b and Supplementary Figs. 4, 5a).

**Fig. 5:**
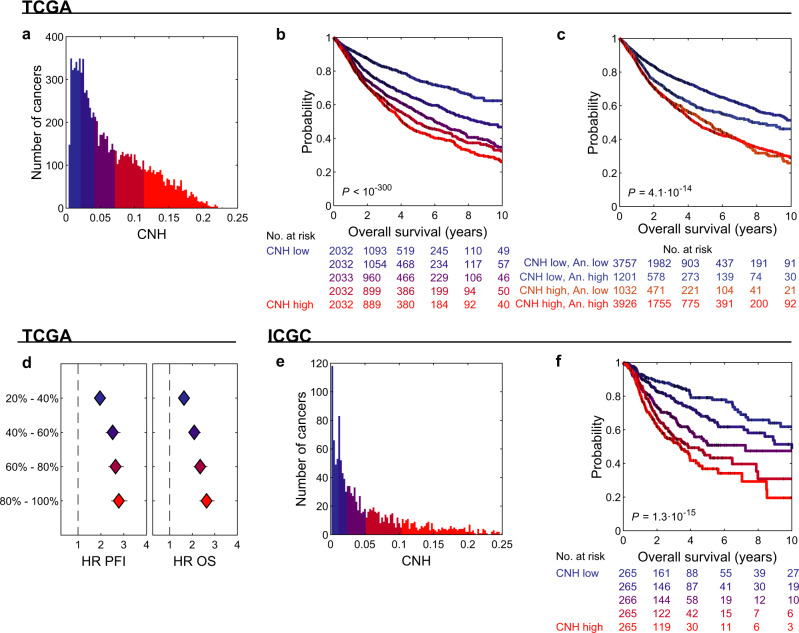
CNH predicts pan-cancer survival rates. **a** Distribution of copy number heterogeneity (CNH) of 10,208 primary cancers with copy number data in TCGA. **b** Kaplan–Meier plots of overal survival (OS) of patients split into five groups of equal size based on rank-ordered CNH of their primary cancer. The most homogeneous and most heterogeneous groups are compared by the two-sided log-rank test. **c** Kaplan–Meier plots of OS of CNH and aneuploidy score low/high cross groups. CNH and aneuploidy score are each split at the median value. The CNH-high, aneuploidy-low and CNH-low, aneuploidy-high groups are compared by the two-sided log-rank test. **d** Hazard ratios of CNH groups for progression-free interval (PFI, left panel) and OS (right panel). Hazard ratios were calculated using Cox proportional-hazard model for each group relative to the 20% most homogeneous patients. Error bars represent 95% confidence intervals of hazard ratios as given by the 2-sided Wald test. The colours in **b** and **d** correspond to the CNH groups as indicated in (**a**). **e** Distribution of CNH of 1326 primary cancers with copy number data in ICGC. **f** Kaplan–Meier plots of OS of patients split into five groups of equal size based on rank-ordered CNH of their primary cancer. The most homogeneous and most heterogeneous groups are compared by the two-sided log-rank test. The colours in **f** correspond to the CNH groups as indicated in (**e**). Source data are provided as a Source Data file.

The ITH of the cancer genomes of all patients can be compared on a continuous quantitative scale by CNH. We explored the variation in CNH and survival in a cancer-type agnostic setting, including 10,208 primary cancers with copy number data in TCGA. The median CNH of all cancers is 0.051 and ranges from 0.003 to 0.23 (Fig. 5a). We split patients rank-ordered by the CNH of their primary cancer in five groups of equal size and determine the survival rates for each group. We find that survival rates decrease monotonically with increasing CNH (Fig. 5b). Importantly, we find that it is heterogeneity at the CNV level that determines the poor prognosis, not the mere presence of CNVs (Fig. 5c). CNH also outperforms other measures of ITH (ABSOLUTE ITH^38^, MATH^11^, PyClone^10^, EXPANDS^12^ and S-score^19^), as well as the overall amount of CNVs^35^, in predicting survival across cancers (Supplementary Fig. 6). Hence, CNH accurately predicts survival across cancer types, with a hazard ratio of 2.6 (2.3–3.0) for the 20% patients with the most heterogeneous malignancies compared to the 20% patients with the most homogeneous malignancies (Fig. 5d). Importantly, also in an independent dataset from the International Cancer Genome Consortium (ICGC), we find that CNH explains survival rates across cancers (Fig. 5e, f)^1^.

**Fig. 6:**
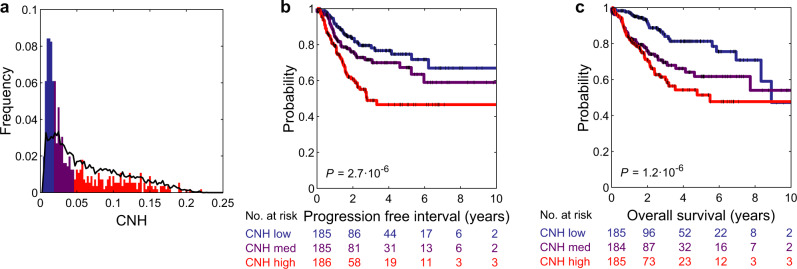
Distribution of CNH and survival curves of microsatellite-instable cancers. **a** Distribution of copy number heterogeneity (CNH) for 558 microsatellite-instable (MSI) tumours (bars) versus all 10,208 primary cancers (line) in TCGA. Kaplan–Meier plots of progression-free interval (**b**) and overall survival (**c**). Patients with MSI tumours are split into three groups of equal size based on rank-ordered CNH in the survival analysis. The most homogeneous (blue) and most heterogeneous (red) groups are compared by the two-sided log-rank test. Source data are provided as a Source Data file.

**Fig. 7:**
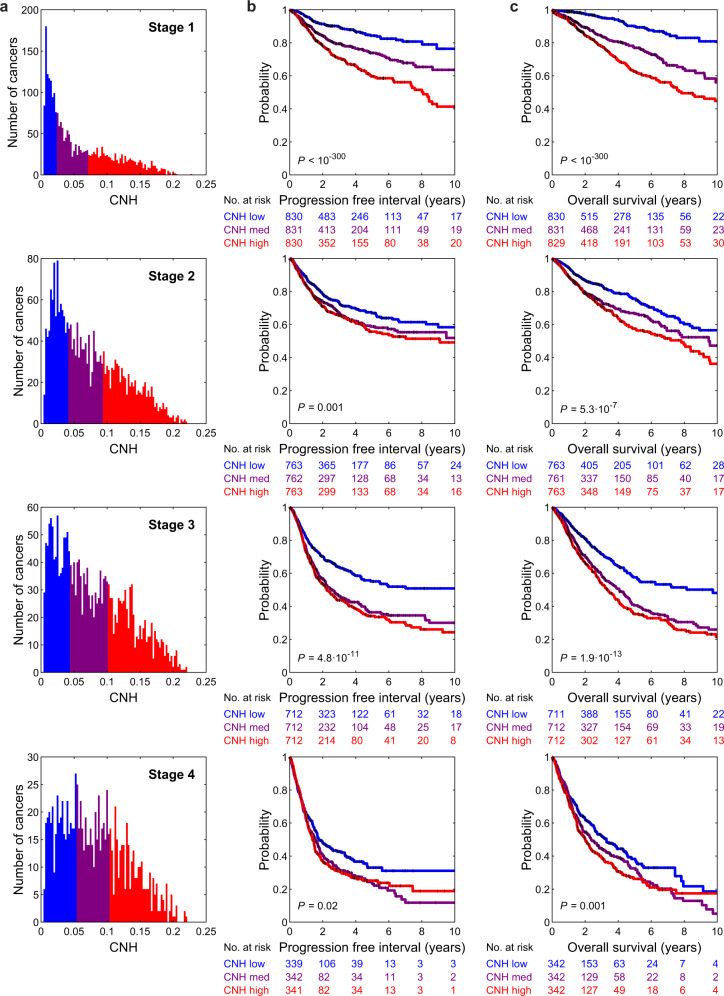
CNH increases with stage and is predictive within each stage. **a** Distribution of copy number heterogeneity (CNH) per stage, for 7792 primary cancers in TCGA with known stage and copy number data. Kaplan–Meier plots of progression-free interval (**b**) and overall survival (**c**). Per stage, patients are split into three groups of equal size based on rank-ordered CNH in the survival analysis. The most homogeneous (blue) and most heterogeneous (red) groups are compared by the two-sided log-rank test. Source data are provided as a Source Data file.

**Fig. 8:**
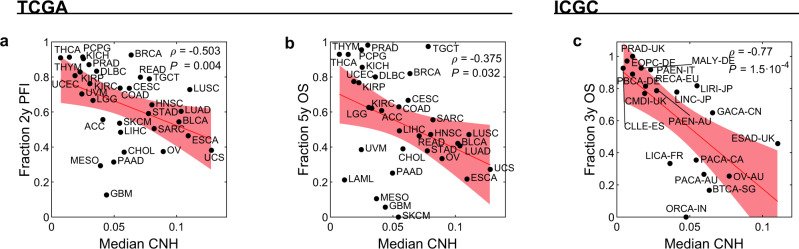
CNH predicts survival rates of cancer types. Median copy number heterogeneity (CNH) versus the fraction of patients with 2-year progression-free interval (PFI, **a**) and 5-year overall survival (OS, **b**) for each cancer type in TCGA. Spearman’s rank correlation is reported. The red line (shade) is a linear fit (95% confidence interval). **c** Median CNH versus the fraction of patients with 3-year OS for each study in ICGC. Spearman’s rank correlation is reported. The red line (shade) is a linear fit (95% confidence interval). Source data are provided as a Source Data file.

The variation in CNH and the relation to survival might be confounded by other tumour or clinical characteristics, such as cancer type, stage of the disease and MSI status. We address the relation of CNH and survival in the context of these three observables. MSI tumours are thought to be driven by defects in the DNA repair machinery, rather than by chromosomal instability. Nevertheless, also for this molecularly defined subgroup, patients with the most heterogeneous cancers have the poorest prognosis (Fig. 6). For 7972 cancers, the stage is reported in TCGA^37^. We find that CNH increases with stage (median CNH per stage: stage I, 0.038; stage II, 0.062; stage III, 0.067; stage IV, 0.075; Fig. 7a). The positive correlation between CNH and stage suggests that heterogeneity in copy numbers fosters progression. Nevertheless, within each stage, the survival rates decrease with increasing CNH (Fig. 7b, c), showing that differences in survival rates cannot be explained simply from the stage at diagnosis. Malignancies from different cancer types vary in CNH (Supplementary Fig. 4). Strikingly, the variation in CNH between cancer types correlates to the variation in survival rates of the cancer types (Fig. 8). CNH thus provides a potential molecular explanation for the remarkable differences in survival rates between cancer types. Finally, multivariate analysis, including CNH, aneuploidy score, genome doubling, cancer type, MSI status, stage, age, gender and mutation status of *TP53*, *KRAS, MYC, PTEN, VHL, PIK3CA, APC* and *BRAF*, shows that CNH is a pan-cancer prognostic biomarker for both the PFI and OS (Supplementary Table 1 and Supplementary Fig. 5b–d).

## Discussion

The CNH measure introduced here allows direct comparison of all cancer genomes on a quantitative and comprehensive scale. Our analyses of ITH in the pan-cancer TCGA and ICGC cohorts suggest that heterogeneity at the copy number level is a universal predictor for survival of cancer patients, that emerges from ongoing chromosomal instability in interplay with cell intrinsic and external selective pressures.

The ITH of a cancer can be accurately inferred from a single copy number measurement, using the CNH measure introduced in this paper. We extensively validated the accuracy and robustness of our method using simulations, single-cell karyotype sequencing, multi-region CNV data, re-sampling from the same tumour and benchmarking to other ITH methods. However, we note that a possible limitation of single-sample measures is sampling bias^39^.

Single-sample CNV measurements can be obtained from stored FFPE material at relatively low costs^40^, and we indeed demonstrated that CNH can be successfully inferred from FFPE-derived data. Our method thus has potential to readily translate into the clinic. However, we note that measurements from FFPE material are typically more noisy than measurements from fresh frozen material, and that the survival analyses in the present study are all based on fresh frozen material.

Analyses of gene-expression data and live imaging of cell divisions in organoids revealed that ongoing chromosomal instability importantly contributes to CNH. Ongoing chromosomal instability implies that continuously new karyotypes are generated. Hence, our results suggest that ITH in CNVs cannot be properly understood as, and quantified by, coexistence of a few clones, as has been reported for ITH determined from SNVs^10,12^. The relation between ITH in CNVs and ITH in SNVs could be an interesting topic for future studies.

Chromosomal instability has been shown to be a negative predictor of survival in several cancer types^28^. Aneuploidy, immune escape and inflammation have been proposed as mechanisms through which chromosomal instability results in poor prognosis^20,41^. Our results provide an alternative explanation: chromosomal instability generates genomic diversity in the malignant cell population on which natural selection acts.

Although we find an important relation between CNH and chromosomal instability, we stress that these are not identical observables. Chromosomal instability is a process at the cell level; CNH is a state at the level of the malignant cell population. While chromosomal instability increases ITH, other cell intrinsic and external selective pressures can reduce the heterogeneity, e.g. differences in fitness between malignant cells and immune suppression. Further understanding of the relation between CNH, selection and chromosomal instability might be obtained by sampling at multiple time points and interpreting the results with evolutionary models. For example, in a recent publication by Minussi et al., it was concluded from extensive karyotyping of single cells from triple-negative breast cancers combined with mathematical modelling, that ongoing chromosomal instability persists during expansion of primary cancers^42^, in line with our conclusions.

CNH increases with stage, stratifies patients for survival within most cancer types and across molecular subgroups and explains why some cancer types have a dismal prognosis compared to other cancer types. The covariance of CNH with stage, and also grade, reduces the independent predictive value of CNH in multivariate analyses. On the other hand, the covariance of CNH with stage and survival implies that progression of disease can be assessed from the molecular properties of the primary cancer. CNH could thus provide a unifying molecular explanation for variations in survival time of patients, from a cancer evolution perspective.

## Methods

### Data sets

TCGA—Segmented copy number data, gene-expression data, mutation data and MSI data of TCGA were downloaded from http://gdac.broadinstitute.org/. In all analyses, only samples from a primary tumour or a primary blood-derived cancer were included (*n* = 10,578 with copy number data). We applied noise filtering based on segment noise (described below), filtering out 370 samples (*n* = 10,208 that pass quality control).

The copy number data from the ‘genome_wide_snp_6-segmented_scna_hg19’ files were used. The MSI status of each sample was obtained from the ‘patient.microsatellite_instability_test_results.microsatellite_instability_test_result.mononucleotide_and_dinucleotide_marker_panel_analysis_status’ variable from the ‘Merge_Clinical’ data files. For the gene-expression data, the RSEM from the ‘illuminahiseq_rnaseqv2-RSEM_genes’ files was used. Mutation data were obtained from the ‘Mutation_Packager_Oncotated_Calls.Level_3’ files. Mutations annotated as SNPs in columns ‘dbSNP_Val_Status’ and as Silent in columns ‘Variant_Classification’ were excluded.

Tangent-normalized probe copy numbers and raw SNP probe data were downloaded from https://portal.gdc.cancer.gov/legacy-archive. Probe intensities from .CEL raw data files of SARC, UCEC, LGG and LAML were processed using Affymetrix Power Tools and allele-specific copy numbers were calculated using HAPSEG (version 1.1.1) and ABSOLUTE (version 1.0.6).

Genome doubling, aneuploidy score and mutational load, and ploidy, purity and ITH, as determined by ABSOLUTE using copy number and mutation data, were obtained from Thorsson et al.^38^. Patient progression and survival times, disease stages, grade of disease, age and gender were obtained from Liu et al.^37^. Chromothripsis determined per chromosome from whole-genome sequencing was obtained of 759 TCGA patients from Cortes-Ciriano et al.^43^.

BriTROC-1—The ovarian cancer dataset was obtained from the corresponding author and contains 253 primary and relapsed ovarian cancer samples from 132 patients in the British Translational Research Ovarian Cancer Collaborative (BriTROC-1) cohort^25^ (Supplementary Fig. 1d, e).

TRACERx—The multi-region non-small-cell lung cancer data from TRACERx consist of 303 samples from 100 patients and the copy number data were obtained from Jamal-Hanjani et al.^18^ (Supplementary Fig. 1a).

CAIRO2—Copy-number data of 96 patients from the CAIRO2 trial^44^ were measured with arrayCGH by Haan et al.^45^ (available from expression omnibus (GEO), accession code GSE36864) and shallow–whole-genome sequencing by Smeets et al.^46^ (available from EGA, accession code EGAS00001002617). We used these data for technical validation of our method (Supplementary Fig. 2i).

Single cells—Single-cell karyotype sequencing of seven samples from colorectal cancer (CRC) patients was performed^24^. Acquisition of patient samples was conducted in accordance with the Declaration of Helsinki with the approval of the Amsterdam UMC, VU University Medical Ethical Testing Committee (2016.254-NL57226.029.16 and 2017-302(A2018)). All patients provided written informed consent. In addition, single-cell data from four CRC samples and 12 healthy colon and CRC-derived organoids were obtained from Bolhaquiero et al.^24^.

Organoids for live imaging—Imaging of four oesophageal cancer-derived organoid lines (CAM277, CAM408, CAM479 and CAM486) and two ovarian cancer-derived organoid lines (HGS-1 and HGS-3.1) was performed as described previously^24^. Briefly, the percentage of cell divisions with chromosomal missegregation was recorded following overnight time-lapse imaging of organoids expressing H2B-Dendra. Genomic DNA was isolated from each organoid line and measured with Illumina SNP chips GSA v3. GenomeStudio was used with standard settings to obtain copy numbers from the SNP data. In addition, live-imaging data of 12 CRC and normal intestinal organoid lines, with the corresponding single-cell karyotype sequencing data to calculate CNH, were obtained from Bolhaquiero et al.^24^ (Supplementary Fig. 3d).

ICGC—Processed copy number somatic mutation data files were downloaded to the Collaboratory cancer cloud. Segmented copy number values were obtained from the ‘TCNExact’ variable. Cancer types were defined according to ICGC project code. Patient OS data were obtained from the ICGC data portal: https://dcc.icgc.org/releases/PCAWG/clinical_and_histology. Variables ‘donor_survival_time’ (OS) and ‘donor_vital_status’ (censoring) from the file ‘pcawg_donor_clinical_August2016_v9.xlsx’ were used. A total of 1326 patients from 19 studies had copy number and survival data, and were not part of TCGA (Figs. 5e, f and 8c).

PyClone and EXPANDS—Single-sample measurements of ITH by PyClone^10^ and EXPANDS^12^ of 1152 and 1095 primary cancers, respectively, of pan-cancer TCGA data, were obtained from Andor et al.^9^.

### Copy-number heterogeneity (CNH)

Input—CNH is calculated from a single segmented whole-genome copy number profile. The copy number data must have been obtained from a bulk sample, i.e. not from a single cell.

Calculation—The segmented copy number profile is first normalized by dividing through the mean copy number. The resulting relative copy number ${r}_{i}$ of segment $i$ with width ${w}_{i}$ depends on the absolute copy number ${q}_{i}$ of segment $i$, on the average sample ploidy$\,\tau\!\!:$

1 $$\tau =\frac{\mathop{\sum}\limits_{i}{w}_{i}{q}_{i}}{\mathop{\sum}\limits_{i}{w}_{i}}$$

and on the purity of malignant cells in the sample $\alpha$:

2 $${r}_{i}=\frac{\alpha {q}_{i}+2(1-\alpha )}{\alpha \tau +2(1-\alpha )}$$

To derive the CNH from the measured relative copy numbers using this expression, we note that ${q}_{i}$ are integers for samples without heterogeneity, and deviations from integer values ${d}_{i}\!\!:$

3 $${{d}_{i}={\rm{|}}q}_{i}-{{\mathrm{round}}}\left({q}_{i}\right){\rm{|}}$$

reflect heterogeneity in the sample. The rationale to find the CNH is hence minimization of the deviations of ${q}_{i}$ from integer values:

4 $${{\mathrm{CNH}}}={{\min }}_{\alpha ,\tau }\left(\frac{\mathop{\sum}\limits_{i}{d}_{i}{w}_{i}}{\mathop{\sum}\limits_{i}{w}_{i}}\right)$$

Minimization over tumour purity and ploidy is done as these are typically unknown sample properties. Using the experimentally obtained relative copy numbers ${r}_{i}$ and varying tumour purity ($\alpha =0.2, 0.21,\ldots, 1$) and tumour ploidy ($\tau =1.5, 1.55, \ldots, 5$) over biologically relevant ranges, one finds the CNH of a sample by solving Eqs. (1–4).

Samples with known purity or ploidy—A sample’s purity or ploidy can be (approximately) known from independent measurements. In that case, minimization in Eq. (4) might be restricted to a specific range or fixed value for each sample to find CNH. For example, in the analysis of the Ovarian cancer dataset, we restricted the purity to a narrow range of 10% around the reported purity for each sample (Supplementary Fig. 1d, e). The ploidy and purity determined by ABSOLUTE were used to calculate ‘CNH ABSOLUTE’ (Supplementary Fig. 6a, c).

Robustness of inference—Inference of CNH can be performed, also if a sample contains no or few CNVs. With few CNVs, a low value of CNH will be found (Supplementary Fig. 3b). Furthermore, noise in the CNV data affects CNH, but the inference can be optimized to handle noise as detailed below and in Supplementary Fig. 2. Only if all CNVs occur at the same frequency, a scenario we did not come across, the inference is not applicable. CNH is designed as a genome-wide measure. Although segments of the genome that are subject to high noise can be excluded, in general, we recommend against applying the CNH method to single genomic segments or chromosome arms.

Invariance of CNH—CNH is defined as the average segment distance to integer values of the absolute copy number profile. A translation of the absolute copy number profile by an integer value hence leaves CNH invariant. Changing the ploidy by an integer value and adjusting the purity accordingly (purity_new_ = −2/ploidy_new_ + purity_inferred_ + 2/ploidy_inferred_) approximately results in translation of the copy number profile by an integer value. Indeed, CNH is highly correlated to the CNH calculated from the inferred ploidy +/−1 in TCGA data (Supplementary Fig. 1f–i). In these analyses, ploidy is fixed to +/−1 the inferred ploidy, and for the samples for which the transferred ploidy is within the limits we consider biologically relevant ($1.5\le \tau \le 5$) a grid search is done for purity, and the corresponding CNH is reported (*n* = 1884 for ploidy +1; *n* = 4454 for ploidy −1).

Noise filtering—CNH is inferred by default from genome-wide copy number data. It is possible to exclude certain regions of the genome (segments) from the CNH inference, e.g. if local noise is high. The precision at which the copy number value of a segment can be estimated is quantified by the standard deviation of the mean (*σ*_*μ*_) of the distribution of probe/bin copy number values around a copy number segment (Supplementary Fig. 2a). Noise filtering can be applied per segment and on the mean *σ*_*μ*_ of a sample. For the TCGA data, we filtered out samples with 〈*σ*_*μ*_〉 > 0.006 (removing the 370 noisiest samples; average is weighted by segment length) and segments with *σ*_*μ*_ > 0.01 (removing the noisiest segments, composing on average 3.5% of the genome; Supplementary Fig. 2b–f).

### Simulations

Computer simulations were developed to verify the accuracy of the CNH measurement. In these simulations, a malignancy with input CNH *h* is simulated as a collection of 10^9^ cells. The karyotype of the cells in the malignancy is simulated by the following procedure:

1. The segments, with possibly different numbers of copies that together comprise the genome of malignant cells, are either taken from a random sample in the TCGA with a genome fraction altered of more than 50% (Fig. 1d and Supplementary Fig. 1b) or generated as *n* segments of random length (Supplementary Fig. 1c).
2. All malignant cells are first given the same number of copies of each segment (a homogeneous sample).
3. Heterogeneity is introduced by changing the number of copies of each segment randomly by plus or minus one in a fraction *f* of the malignant cells. For each segment, *f* is a random number drawn from a flat distribution between 0 and 2*h, such that $\left\langle f\right\rangle =h$.
4. To include non-malignant cells in impure tumour samples, a fraction 1 − *α* of the malignant cells is replaced by non-malignant cells with a diploid genome.

The average karyotype of the cells in the malignancy is determined and used to infer CNH. For each purity and input heterogeneity, 100 malignancies were simulated and CNH was measured. We find an excellent agreement between the simulated input heterogeneity *h* and the measured CNH of the simulated malignancies.

### Gene expression and CNH

For *n* = 9198 samples of primary cancers, copy number and gene-expression data were both available in TCGA (*n* = 8968 after noise filtering). The expression level of each gene was correlated to CNH using Spearman’s rank correlation. The correlation is done in a pan-cancer setting, and we note that gene-expression patterns vary between cancer types. In total, 18,021 genes were significantly correlated with CNH (*P* < 0.05). The distribution of Spearman’s *ρ* of all genes, however, forms an almost normal distribution (mean = 0.024; 95% confidence interval of mean = 0.022–0.027; standard deviation = 0.1523; 95% confidence interval of standard deviation = 0.1508–0.1513 for the Gaussian fit in Fig. 2a). For the gene ontology analysis, we therefore focused on the genes most positively correlated with CNH in the tail of the distribution (104 genes with *ρ* > 0.42). To determine the gene ontologies associated with negative correlation to CNH on the same number of genes, we selected the 104 most negatively correlated genes. Gene ontologies were determined for all GO terms using the ‘Statistical overrepresentation test’ of PANTHER (http://www.pantherdb.org/; Fig. 2c and Supplementary Fig. 3c)^32,33^.

Chromosomal instability was assessed in each malignancy by the CIN70 signature^28^. The expression of the genes that comprise this signature was normalized per gene by the median expression and summed per malignancy to arrive at the CIN70 score for each malignancy (Supplementary Fig. 3e).

Gene network analysis for co-functionality of genes was performed using GADO^31^. We constructed a list of 100 genes, containing the 20 genes most positively correlated with CNH and 80 randomly picked genes, and used the ‘Function Enrichment’ option in the online tool (https://www.genenetwork.nl/) to build the network in Fig. 2b (genes used for co-functionality analysis are indicated in the source data of Fig. 2). The edge threshold in the network (*Z*-score, representing significance in terms of the number of standard deviations and is equivalent to *P* value) was set to maximum co-functionality (*Z*-score > 13.22). The network in Fig. 2b only contains genes positively correlated to CNH. The *Z*-score can be lowered to 7.06 before this network will contain any of the randomly picked genes.

### Genomic aberrations and CNH

The aneuploidy score quantifies the number of chromosome arms that deviate from diploid and was correlated to CNH using Spearman’s rank correlation (Supplementary Fig. 3a, b)^35^. Genome doubling and mutational load in TCGA data were obtained from Thorsson et al.^38^, and the relation to CNH was assessed by the Wilcoxon’s rank-sum test (Fig. 3a, f) and Spearman’s rank correlation (Fig. 3a, d, e), respectively. The genome fraction altered was defined as the fraction of the genome subject to CNVs. A segment was considered altered if log(*r*) ≤ −0.1 (loss) or log(*r*) ≥ 0.1 (gain).

For *n* = 9656 samples of primary cancers, copy number and mutation data were both available in TCGA (*n* = 9388 after noise filtering). For all genes, samples were grouped as either wild type or mutated. The distributions of CNH between the wild-type and mutated groups were compared using the Wilcoxon rank-sum test.

### Validation of CNH with single-cell data

Single-cell data were generated as described in, and obtained from, Bolhaquiero et al.^24^. Briefly, tissue cuts were lysed in a nuclei Suspension Buffer and stained with 10 μg ml^−1^ Hoechst 34580 (Sigma-Aldrich). The lysed tissue was kept on ice for 1 h after which it was filtered through 70- and 35-μm strainers. Nuclei were sorted on a DB FACS Fusion sorting for G1 state in a 384-well plate containing 5 μl of mineral oil (Sigma) in each well, and stored at −20 °C until further processing for library preparation and sequencing. Library preparation started with a Prot K (Fisher) treatment, after which the genomic DNA was digested with NlaIII restriction enzyme. A cell-specific 8-bp barcode, a 3-bp random molecular barcode, the 5′ Illumina TruSeq small RNA kit adapter and a T7 promoter was ligated. DNA of each cell was pooled and in vitro-transcribed after which it was fragmented and reverse-transcribed, converted to double-stranded cDNA and amplified with PCR. Illumina sequencing libraries were prepared with the TruSeq small RNA primers (Illumina). Libraries were sequenced on an Illumina Nextseq 500 with 75-bp single-end sequencing.

Sequencing reads were aligned to genome build ‘GRCh38.p10’ with bwa (v0.7.12). Quality control was performed with Aneufinder v1.14 with default parameters and as described previously. In addition, cells with reads <20,000 and spikiness >0.25 were excluded. We generate a quasi-bulk copy number by taking an equal amount of reads from each cell for each sample. Quasi-bulk copy number profiles were generated from the pooled reads using QDNAseq^40^. From the quasi-bulk copy number profiles CNH was inferred. Next, we calculate single-cell CNH directly from the single-cell karyotypes (absolute copy numbers) of each sample as follows. We find the minimum consecutive set of segments that comprise the genome of all single cells of a sample. For each segment, we determine the most frequent absolute integer copy number in the sample, and define the local heterogeneity as the fraction of cells that have a value different than the most frequent value. CNH quantified directly from single cells is then defined as the average heterogeneity across segments, weighted by segment lengths and correlated to CNH from quasi-bulk analysis using Spearman’s rank correlation (Fig. 1c).

### Validation of CNH with multi-region data

A multi-region copy number dataset containing 303 samples from 100 patients was obtained from Jamal-Hanjani et al.^18^. All 303 samples in this dataset are bulk samples, i.e. consisting of more than one malignant cell. Hence, CNH can be inferred from the copy number data of each single sample and averaged over all samples from a patient to arrive at an estimate of CNH per patient. In addition, we calculate multi-region CNH from copy number profiles from the different regions of a patient as follows. We find the minimum consecutive set of segments that comprise the genome of all regions measured from a patient. For each segment, we determine the most frequent absolute integer (rounded) copy number in the sample, and define the local heterogeneity as the fraction of regions that have a (rounded) copy number value different than the most frequent value. The multi-region CNH is then defined as the average heterogeneity across segments, weighted by segment lengths. The average CNH of all regions from a patient was correlated to the multi-region CNH using Spearman’s rank correlation (Supplementary Fig. 1a).

### Comparison of CNH to other ITH measures

We compare CNH to other single-sample ITH measures on TCGA data. We obtain ITH according to PyClone (*n* = 1095 primary cancers) and EXPANDS (*n* = 1152 primary cancers) from Andor et al.^9^ and ITH according to ABSOLUTE applied to copy number and mutation data (*n* = 9399 primary cancers) from Thorsson et al.^38^. These three methods use both copy number and mutation data to infer ITH. We calculate *S*-score^19^ from the copy number data of 10,379 primary cancers and MATH^11^ from the mutation data of 9290 primary cancers. In addition, we calculate ITH using ABSOLUTE applied to copy number data only for SARC, LAML, UCEC and LGG (1255 primary cancers). For each of these measures, and CNH, we calculate the concordance-index for predicting OS using the ‘survival’ R package (v3.2.7) and the ‘compareC’ R package (v1.3.1). We find that CNH is significantly better in predicting survival than any of the other methods considered. ABSOLUTE ITH quantifies the fraction of the genome that is sub-clonal, and is therefore the ITH method conceptually most similar to CNH. We correlate each of the ITH measures, including CNH, to ABSOLUTE ITH and find that indeed CNH correlates strongly (and better than the other methods) to ABSOLUTE ITH. We note that performance of ABSOLUTE to measure ITH depends on both CNV and mutation data. Indeed, we find a drop in performance of ABSOLUTE when applied to CNV data only (ABSOLUTE ITH without mutation data has a C-index = 0.53 when applied to data from SARC, LAML, UCEC and LGG. ABSOLUTE ITH, including mutation, has a C-index = 0.55 applied to the same data, and C-index = 0.58 when applied to all data in TCGA). Accurate prediction of ITH by ABSOLUTE hence depends on both CNV and SNV data (Supplementary Fig. 6).

### Statistical analyses and software

The statistical tests used were reported with each analysis and all tests were two-sided. HapSeg (v1.1.1) and ABSOLUTE (v1.0.6) were used to analyze SNParray data. BWA (v0.7.12) was used for read alignment of single-cell data and R-package Aneufinder (v1.14) was used to further analyze single-cell data. Cox proportional-hazard models were constructed with the ‘coxph’ function from the ‘survival’ package (v 3.2.7) and Concordance-indices were determined and compared using ‘CompareC’ function from the ‘CompareC’ package (v1.3.1)^47^. R-version used was R-3.6.3. All other analyses were performed in, and all code was written in MATLAB R2019a.

### Reporting summary

Further information on research design is available in the Nature Research Reporting Summary linked to this article.

## Supplementary information

Supplementary Information

Peer Review File

Description of Additional Supplementary Files

Supplementary Data 1

Supplementary Data 2

Reporting Summary

## Data Availability

TCGA data analyzed in this paper can be downloaded from http://gdac.broadinstitute.org. The BriTROC-1 data are available on: https://bitbucket.org/britroc/cnsignatures/src/master/ and raw data can be obtained by contacting James Brenton, corresponding author of the original publication: James.Brenton@cruk.cam.ac.uk. CAIRO2 data measured with arrayCGH are publicly available from gene-expression omnibus (GEO), accession code GSE36864, and shallow–whole-genome sequencing is available from the European Genome Archive (EGA), accession code EGAS00001002617. Data from TRACERX used in this study are available from Supplementary Appendix 2 of Jamal-Hanjani et al.^18^. Data from ICGC are under restricted access, available from https://dcc.icgc.org/repositories. Data generated in this study: single-cell karyotype of CRC samples and SNP data measured from bulk of the oesophageal and ovarian organoids are available from the EGA, under accession number EGAS00001004702. Source data are provided with this paper.

## Source data

Source Data

## Code availability

MATLAB code to calculate CNH from a segmented copy number profile is available here: https://github.com/dmmiedema/CNH.
