# Supplementary material for: Chromosomal copy number heterogeneity predicts survival rates across cancers
Source: Nat Commun. 2021 May 27;12:3188. doi: 10.1038/s41467-021-23384-6 (PMC8160133; doi:10.1038/s41467-021-23384-6)
Supplement: Supplementary file 1 — Supplementary Information [file 41467_2021_23384_MOESM1_ESM.pdf]

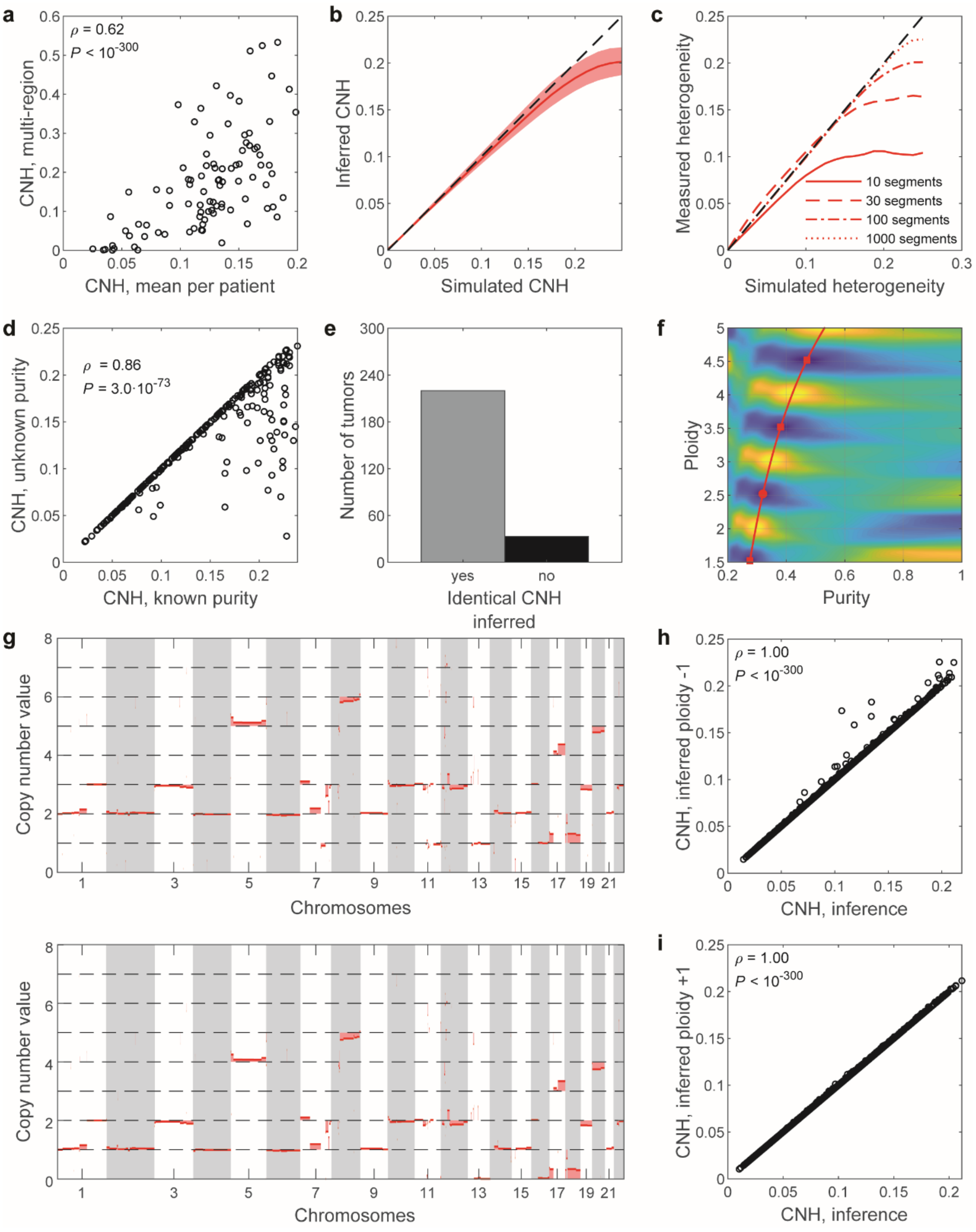

Supplementary Fig. 1 Accuracy of CNH measurement. **a** Copy number heterogeneity (CNH) calculated from TRACERx multi-region copy number data[18]. CNH is calculated from each sample separately and averaged per patient (x-axis) and from direct comparison of the karyotypes from the different regions measured for a patient (y-axis). Spearman's rank correlation is reported with 2-sided *P*-value. **b** Comparison of pre-defined input and measured CNH of simulated copy number profiles. **c** Comparison of pre-defined input and measured CNH of simulated artificial copy number profiles. The artificial copy number profiles consist of 10 (solid line), 30 (dashed line), 100 (dot-dashed line) or 1000 (dotted line) segments. The accuracy of the inferred CNH increases with the number of segments in a copy number profile. **d** Inference of CNH of Ovarian cancers with (x-axis) and without (y-axis) incorporation of purity in the calculation[25]. **e** If the heterogeneity inferred with and without purity differs less than 0.03

the output is considered identical. **f** Example of grid search for purity and ploidy to calculate CNH. The colour indicates the average distance of segments in the absolute copy number profile to integer values (blue=small distance, yellow=large distance). Global and local minima are indicated by red circle and squares, respectively. Red line indicates predicted line of possible local minima. **g** Absolute copy number profiles of the sample in (**f**) corresponding to the global minimum ploidy (top) and this ploidy-1 (bottom). **h**, **i** CNH calculated using inferred ploidy versus CNH from inferred ploidy – 1 (**h**) and inferred ploidy +1 (**i**) for TCGA data. Spearman's rank correlation is reported with 2-sided *P*-value. Source data are provided as a Source Data file.

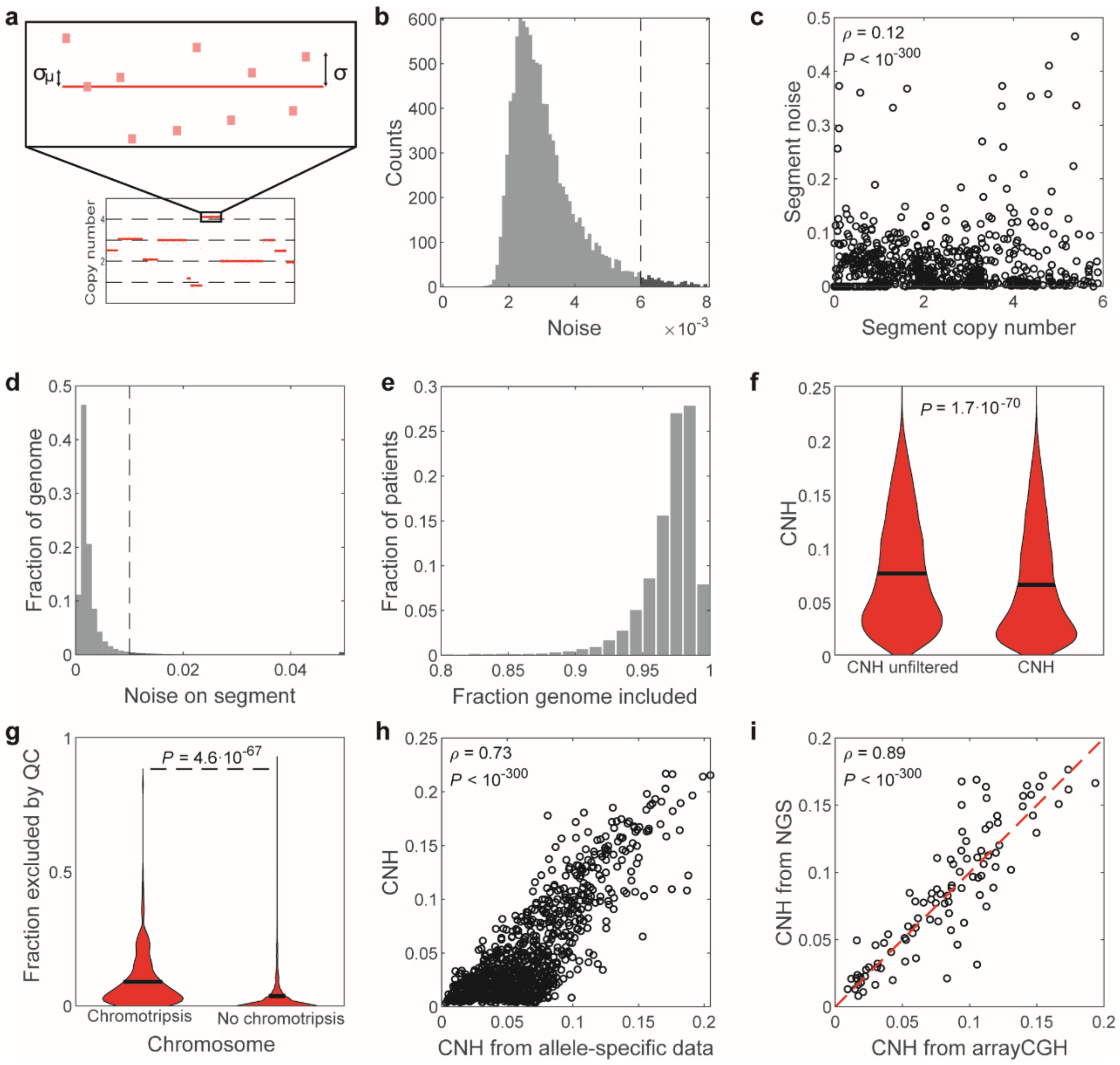

Supplementary Fig. 2 Robustness of CNH measurement. a Noise in segment copy number values translates to noise in copy number heterogeneity (CNH). The precision of a segment copy number value, σ_μ, can be estimated by $\sigma_\mu = \frac{\sigma}{\sqrt{n}}$. With σ the standard deviation of the distribution of the *n* probe/bin copy number values that comprise the segment. **b** Distribution of noise per sample in TCGA. The dashed line indicates the noise threshold: samples with average segment noise above this threshold (n = 370) are excluded from inference of CNH and further analysis. **c** Spearman's rank correlation of noise on a segment to the absolute segment copy number value. The 2-sided *P*-value is reported. **d** Distribution of noise per segment, weighted by segment length. The dashed line indicates the threshold of segments included in the analyses. **e** Fraction of genome included per patient after filtering for noisy segments as indicated in (**d**). **f** Violin plot distribution of CNH across cancers before and after noise filtering. The distributions are compared by the 2-sided Wilcoxon rank-sum test. **G** Fraction of chromosomes excluded by noise filtering for chromosomes with and without chromothripsis[42]. The distributions are compared by the 2-sided Wilcoxon rank-sum test. **h** Spearman's rank correlation between CNH derived from total copy numbers (default; y-axis) and from allele-specific copy numbers (x-axis). The 2-sided *P*-value is also reported. Data shown in (**b-h**) is from TCGA. **i**, Spearman's rank correlation between CNH derived from arrayCGH data and shallow whole-genome sequencing data measured on the same FFPE

stored malignancies of 96 patients from the CAIRO2 trial[43-45]. Source data are provided as a Source Data file.

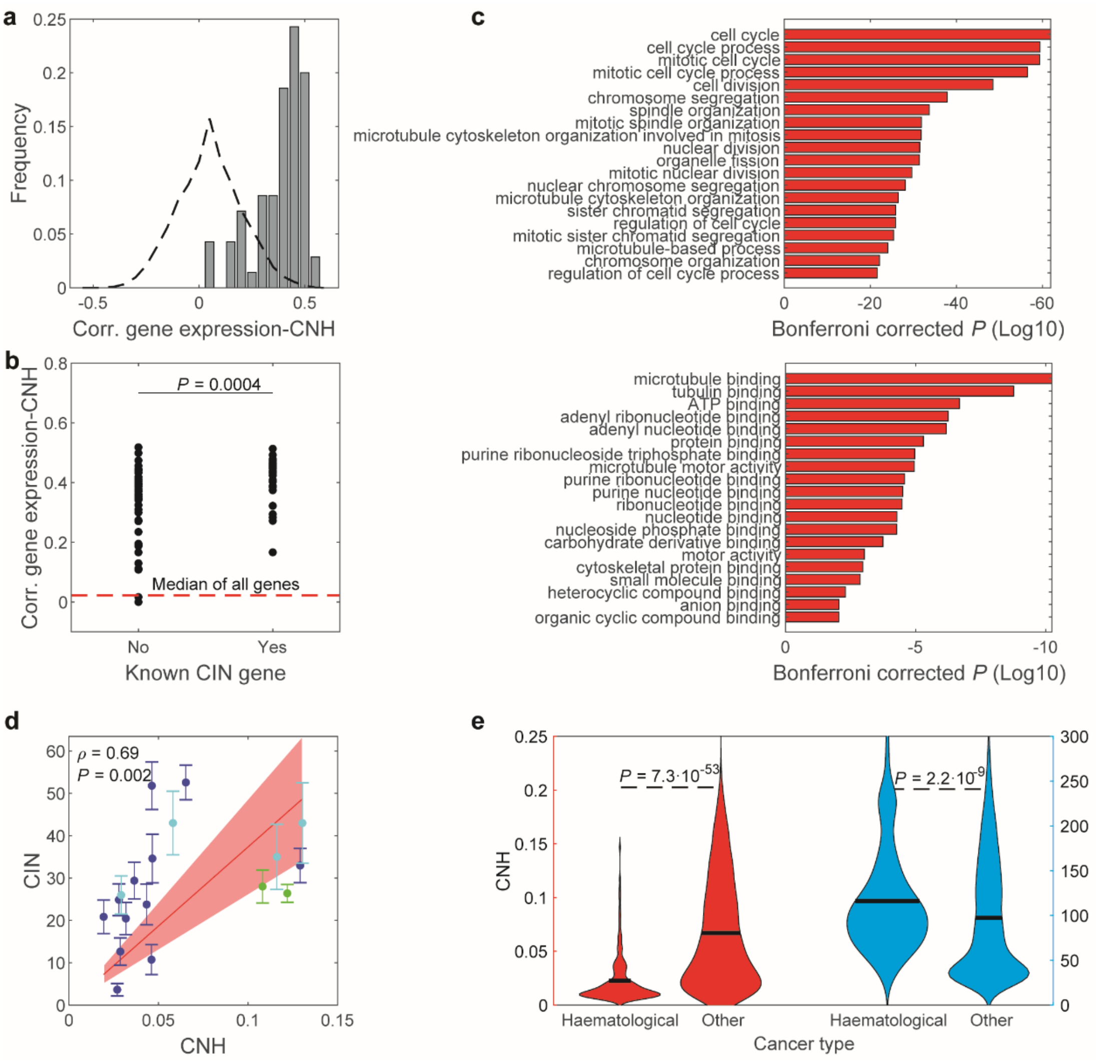

Supplementary Fig. 3 CNH is correlated with chromosomal instability. a Normalized histograms showing Spearman's rank correlation of gene expression to copy number heterogeneity (CNH). The correlation is calculated for genes in the CIN70 signature[28] (bars) and for all genes (dashed line), on 8,968 samples in TCGA which have combined copy number and gene expression data. **b** Spearman's rank correlation of genes in the CIN70 signature to CNH. Genes in the CIN70 signature were annotated as known chromosomal instability (CIN) genes or not. The two groups were compared by the 2-sided Wilcoxon rank-sum test. **c** Most significant biological process (upper panel) and molecular function (lower panel) gene ontologies of the 104 genes most positively correlated to CNH. **d** The percentage of cell divisions that involve chromosomal mis-segregation (y-axis) determined by live imaging of colon (blue), oesophageal (green) and ovarian (cyan) cancers derived organoids is correlated to CNH. The red line (shade) is a linear fit (95% confidence interval) without intercept. Spearman's rank correlation is reported with 2-sided *P*-value. Dots and error bars indicate mean and standard deviations of CIN **e** Distributions of CNH (red) and chromosomal instability according to the CIN70 signature (blue) divided as haematological or other in cancers from TCGA. Distributions are compared using the 2-sided Wilcoxon rank-sum test. Source data are provided as a Source Data file.

Supplementary Fig. 4

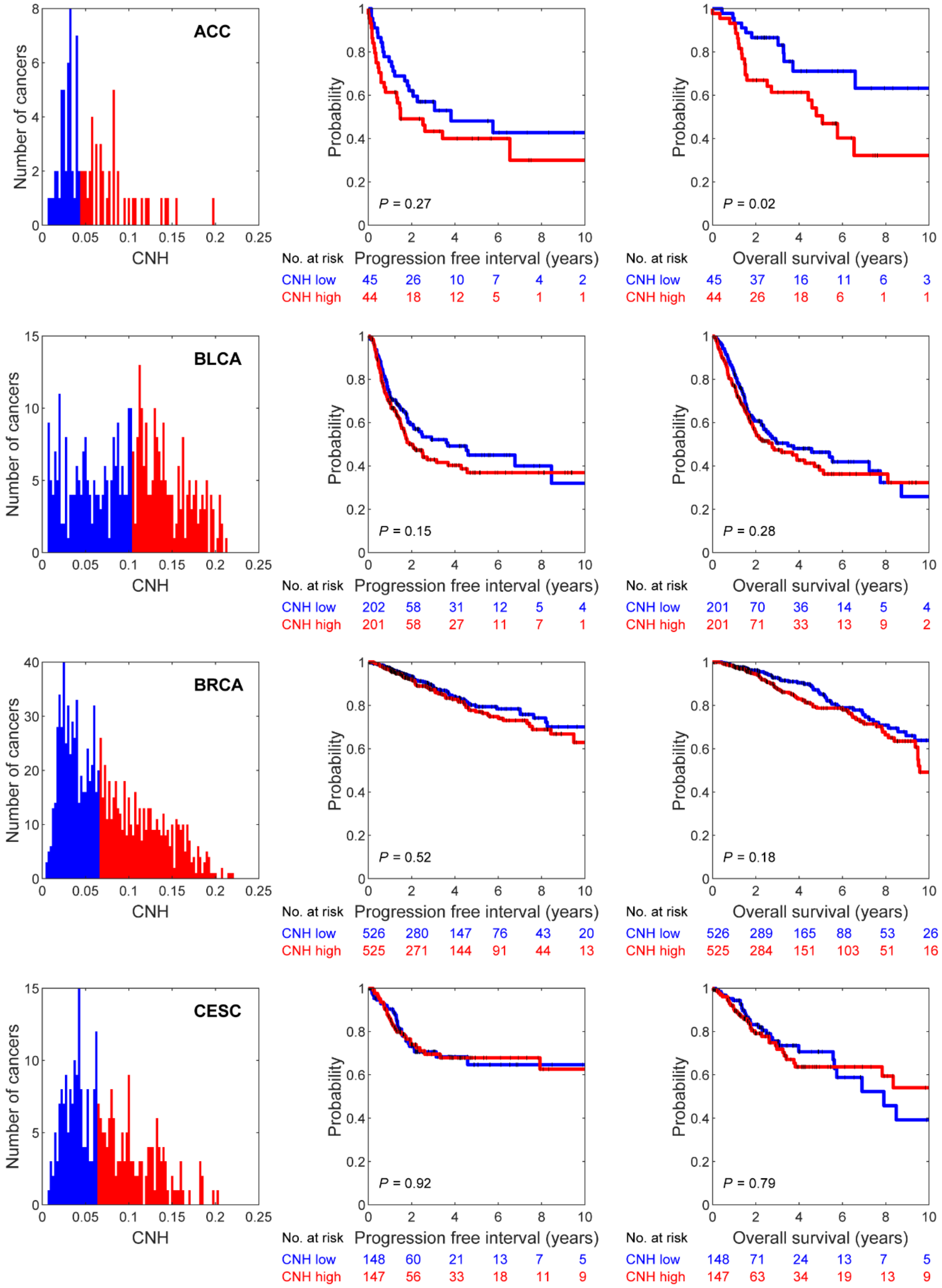

Supplementary Fig. 4 continued

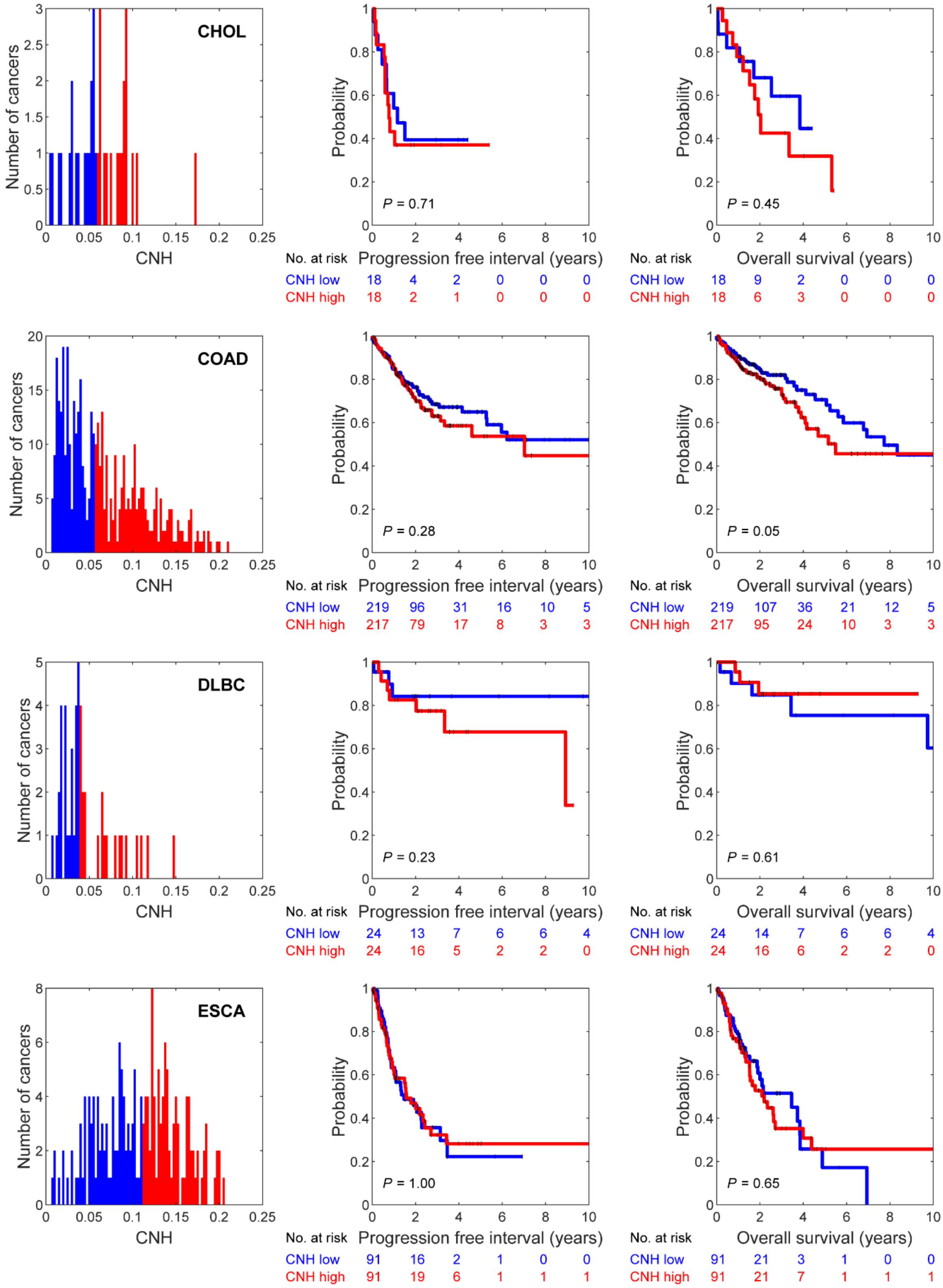

Supplementary Fig. 4 continued

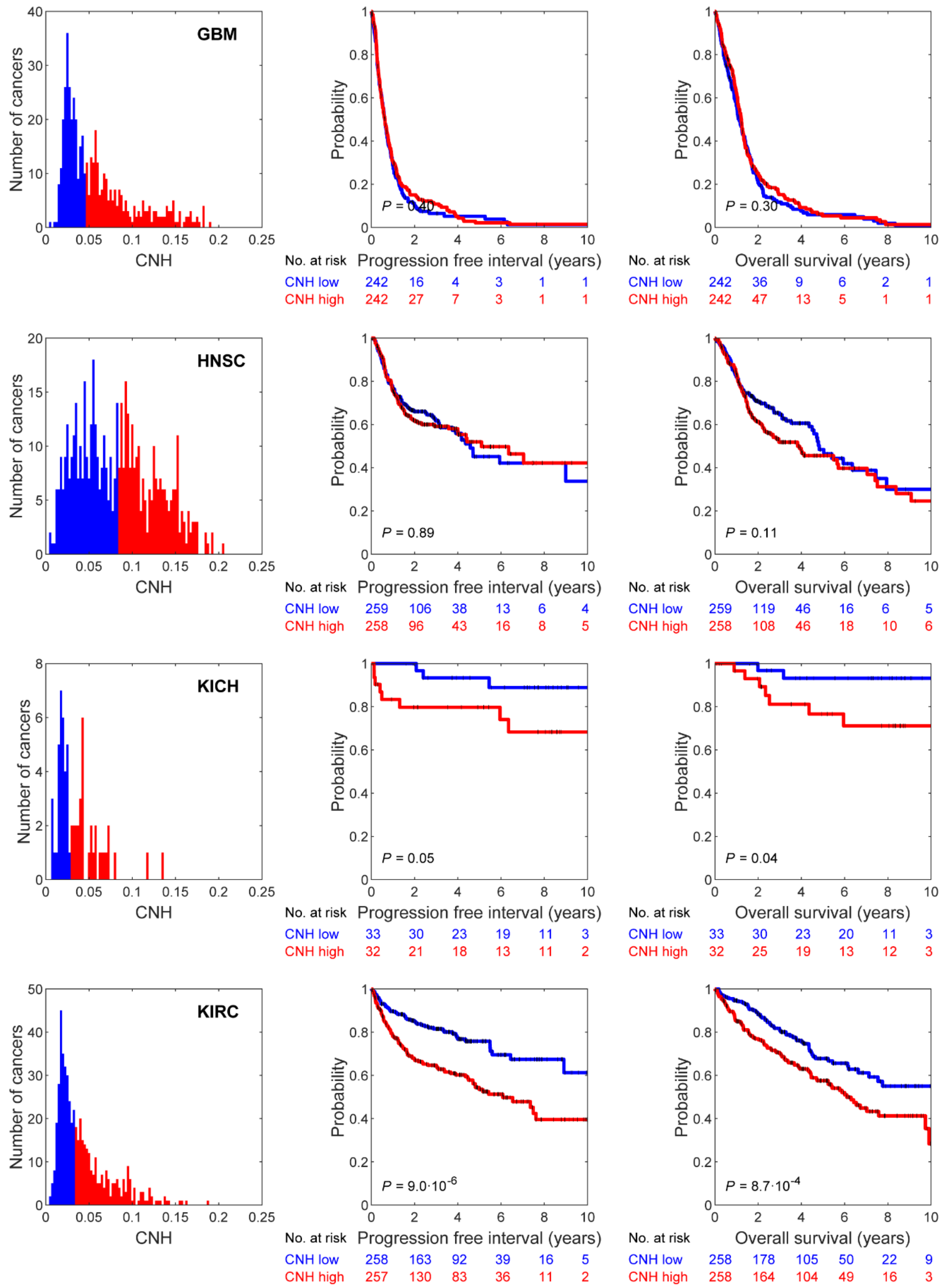

Supplementary Fig. 4 continued

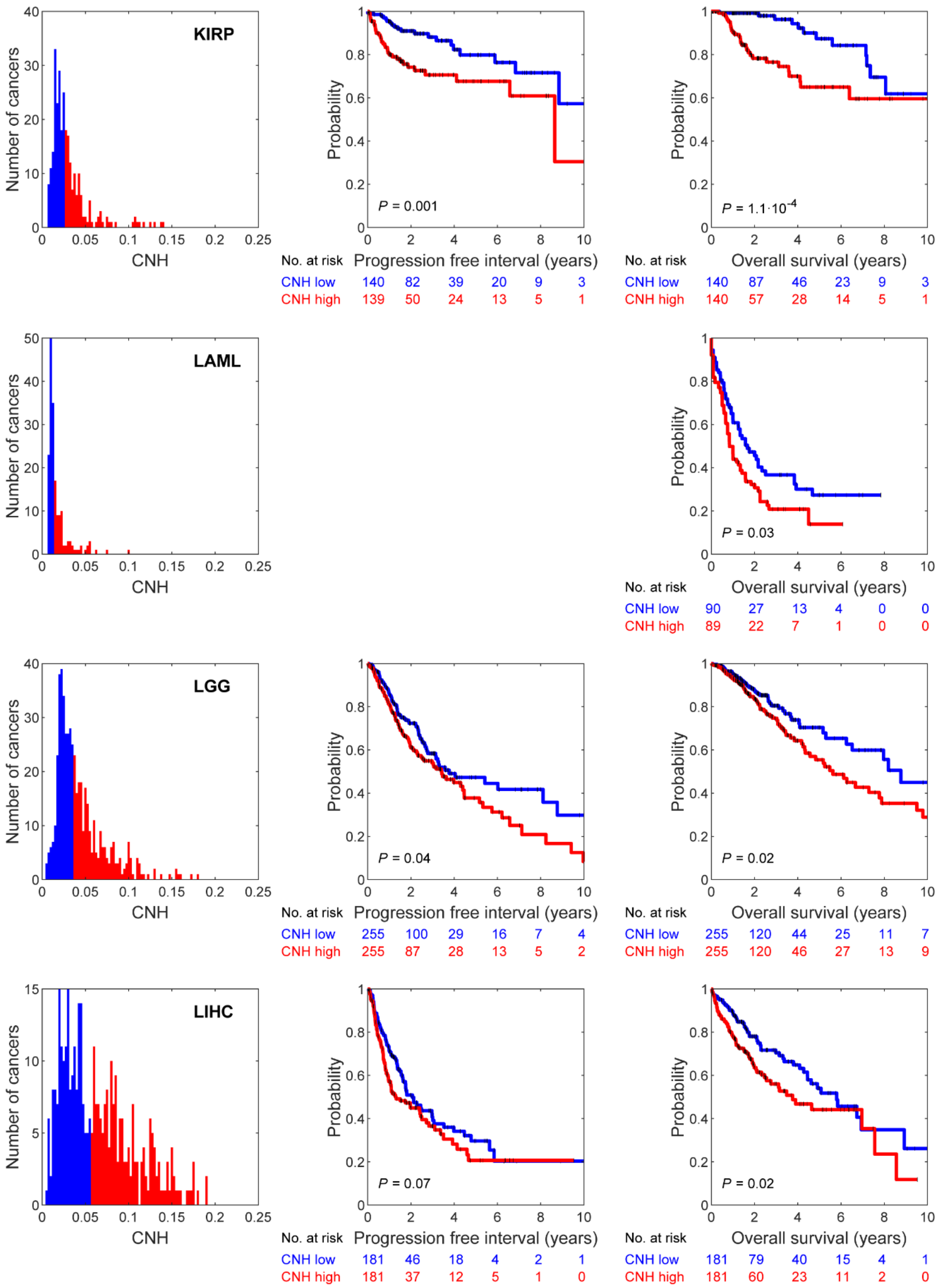

Supplementary Fig. 4 continued

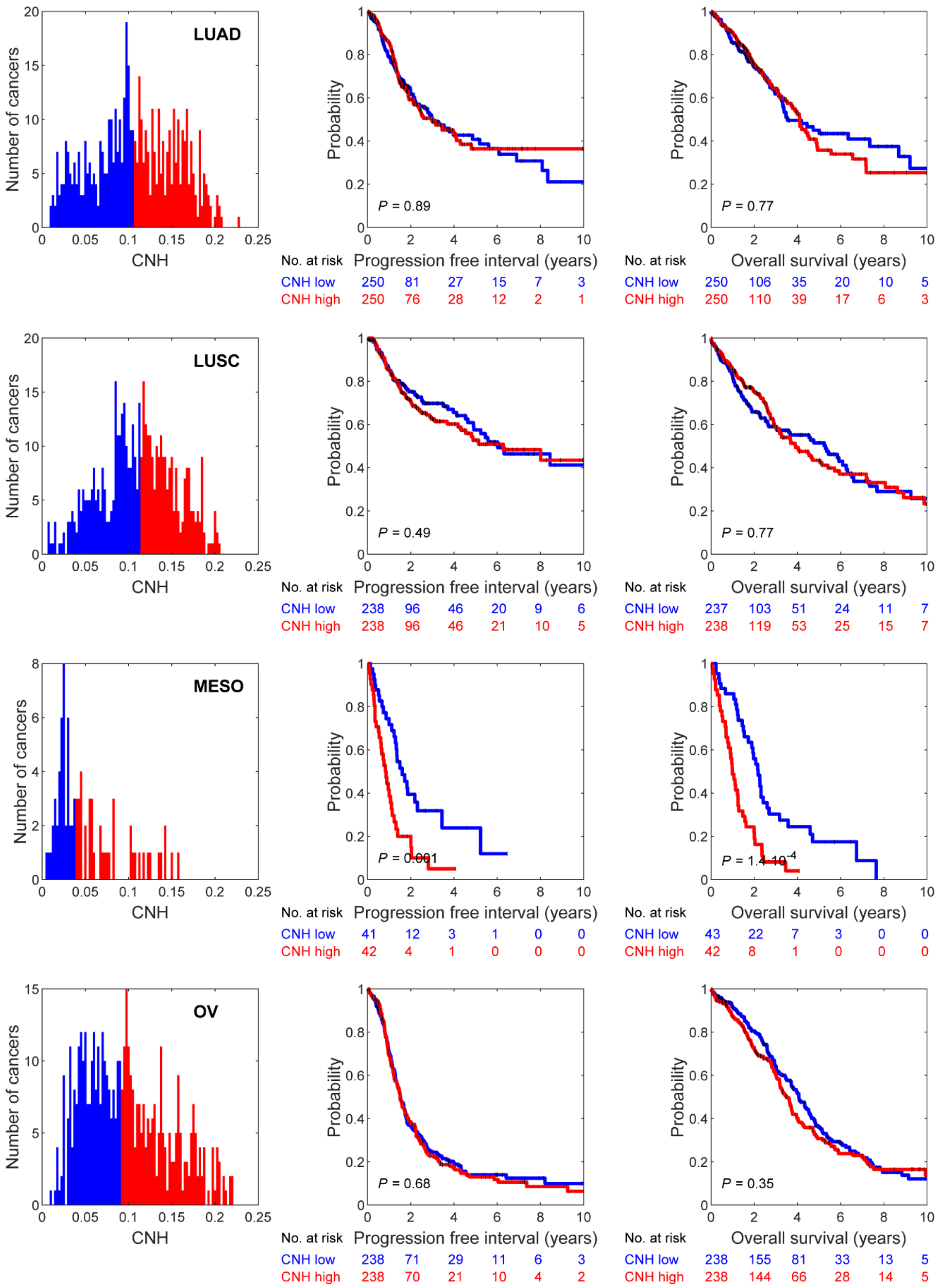

Supplementary Fig. 4 continued

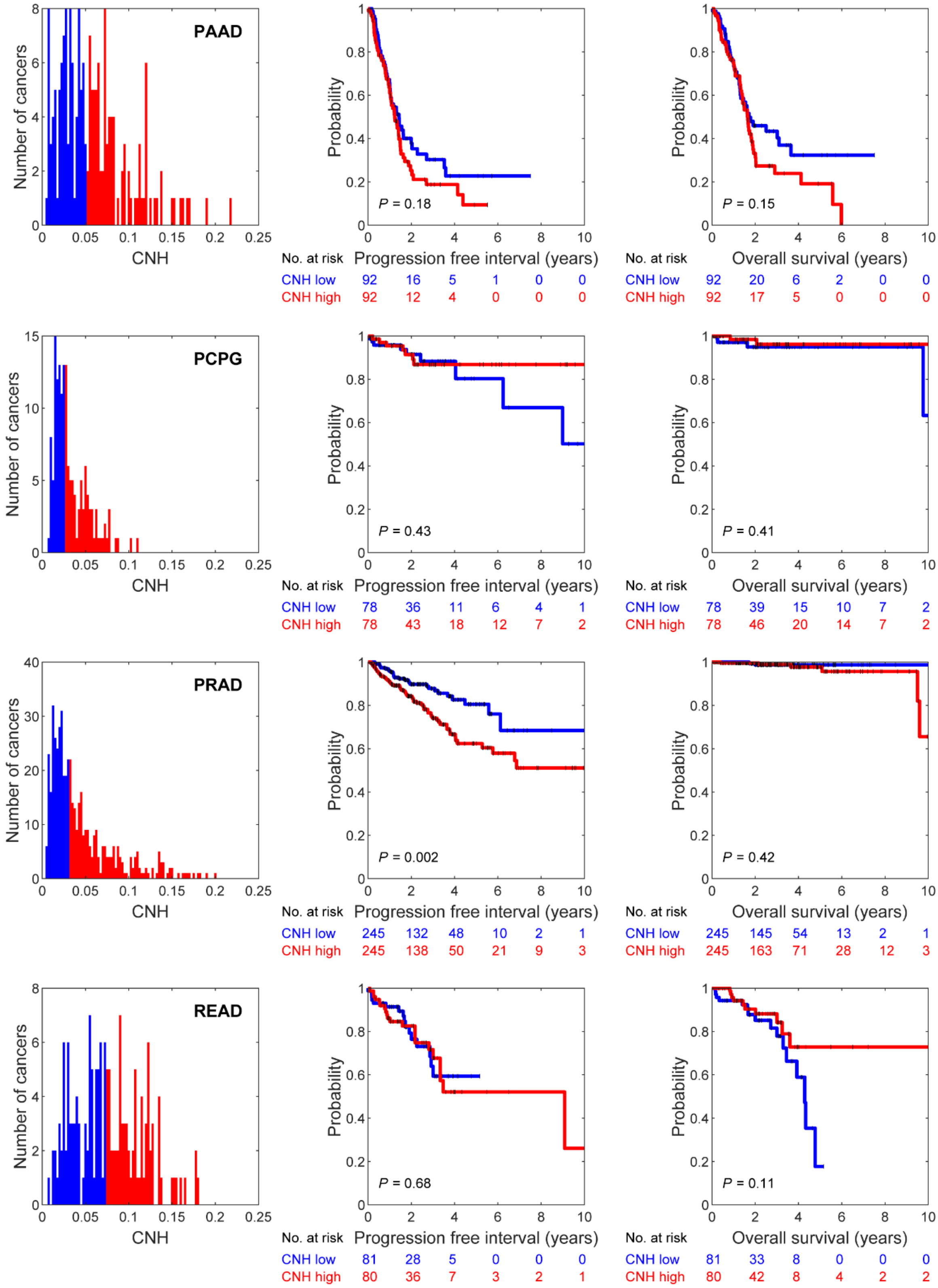

Supplementary Fig. 4 continued

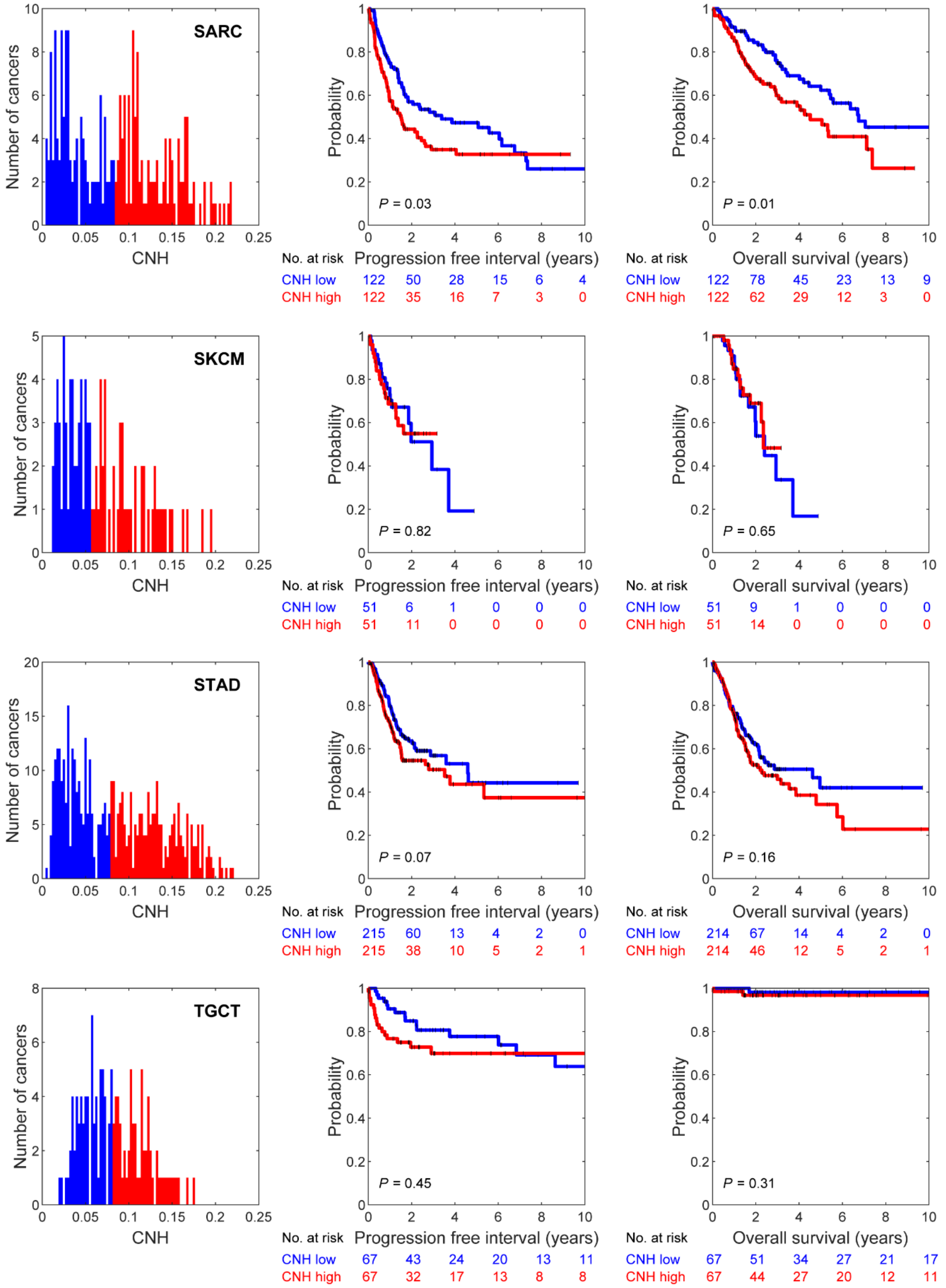

Supplementary Fig. 4 continued

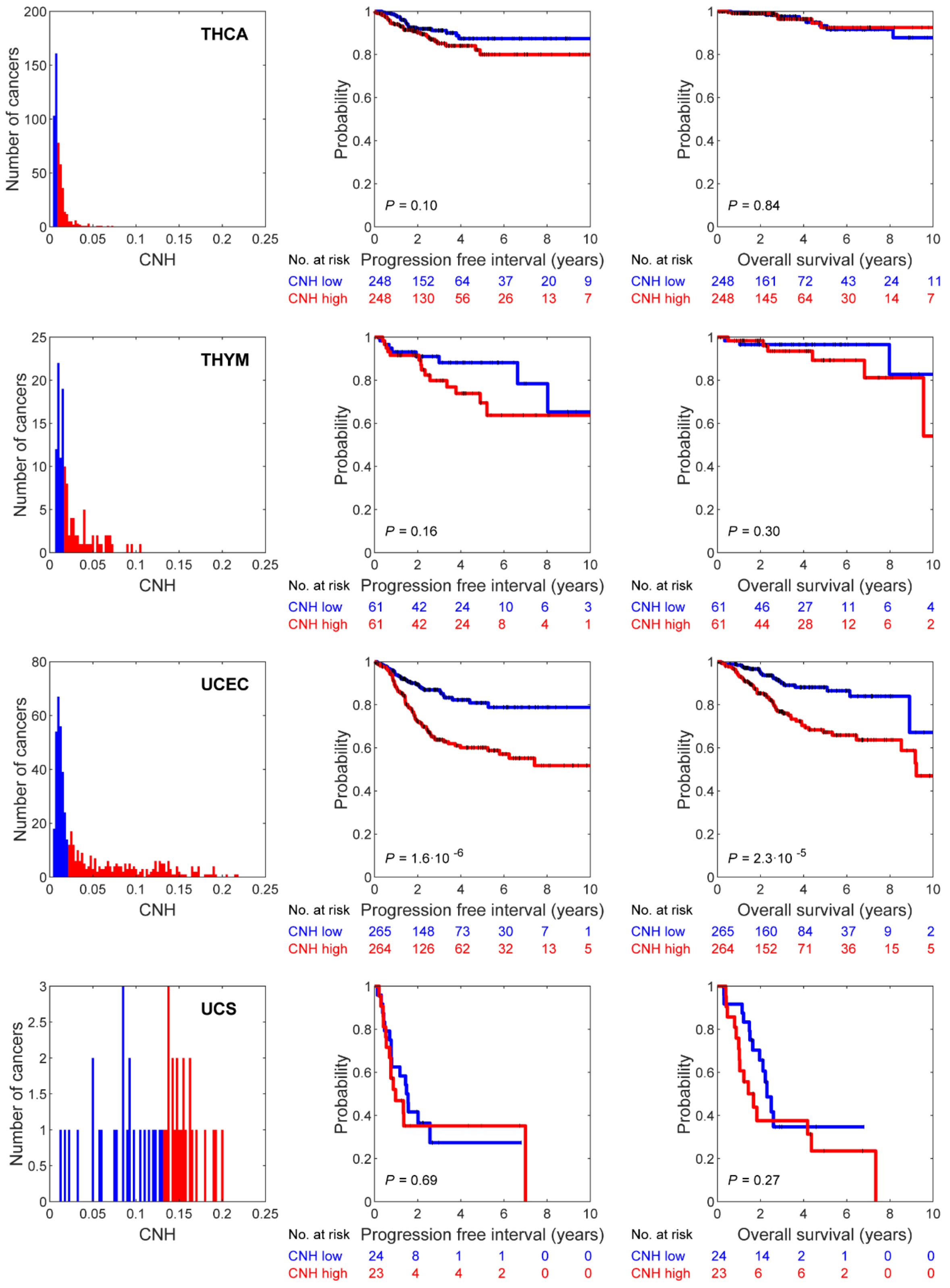

Supplementary Fig. 4 continued

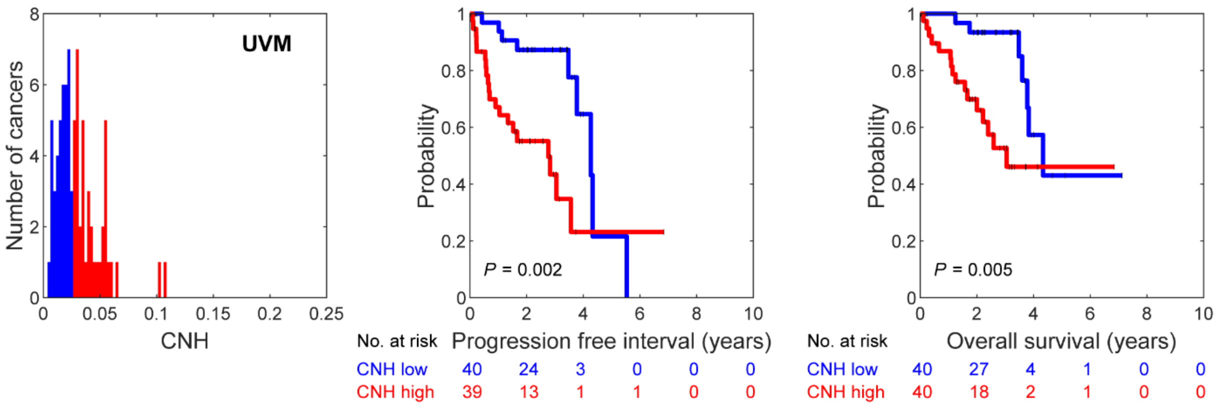

Supplementary Fig. 4 Distribution of CNH and survival curves for all cancer types in TCGA. Left panels: Distribution of copy number heterogeneity (CNH) in each cancer type. Kaplan-Meier plots of progression free interval (middle panels) and overall survival (right panels). Within each cancer type, patients are split in two groups of equal size based on rank-ordered CNH in the survival analysis (homogeneous malignancies, blue; heterogeneous malignancies, red). The *P*-values reported with the Kaplan-Meier curves were calculated using the 2-sided log-rank test. Source data are provided as a Source Data file.

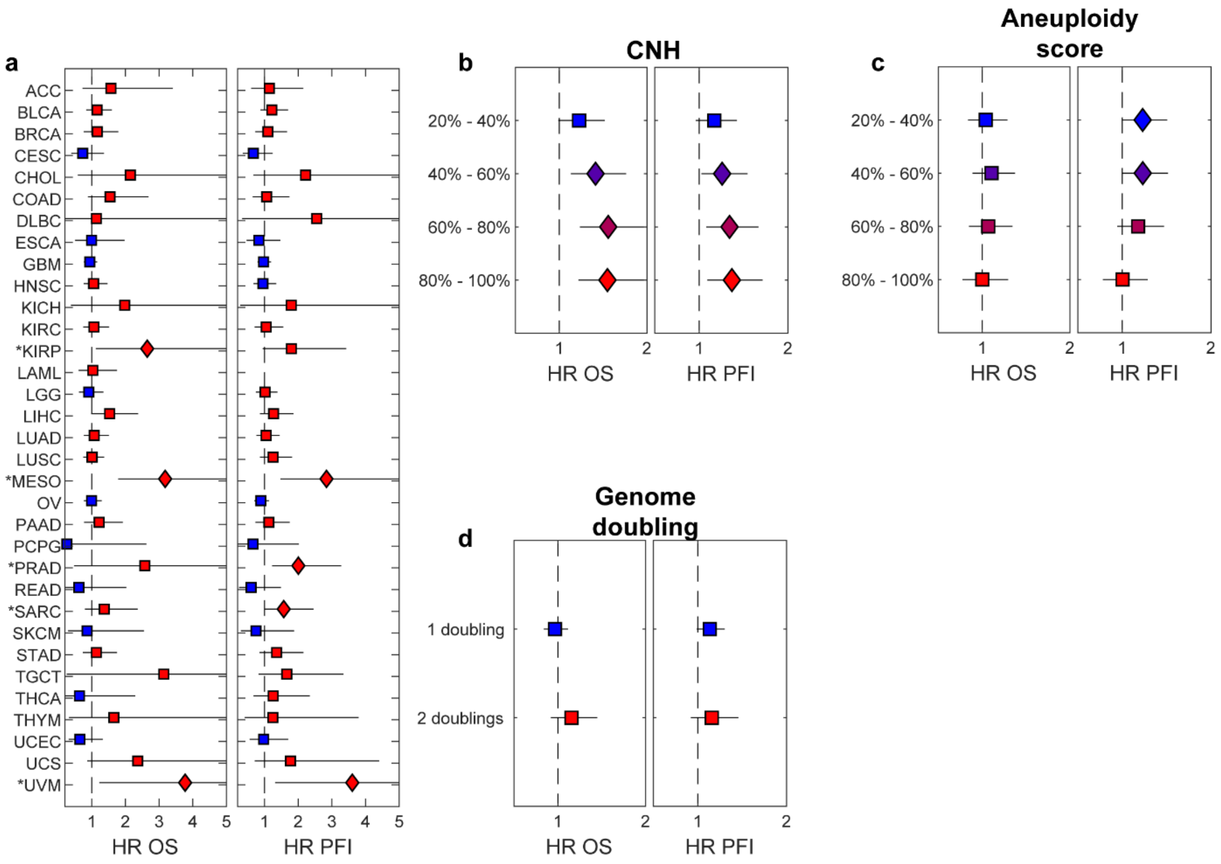

Supplementary Fig. 5 CNH associates with poor prognosis in multivariate analysis. a Hazard ratios (HR) of CNH for overall survival (OS; left panel) and progression free interval (PFI; right panel) of all 33 cancer types in TCGA. Patients were split in two groups of equal size for each cancer type based on the rank ordered CNH of their primary cancer. Hazard ratios were calculated using multivariate Cox proportional-hazards models including: CNH, aneuploidy score, age, gender, stage, grade and MSI status. For each cancer type, only variables that were defined on at least 50% of patients were included in the models. Red (blue) symbol indicates CNH high (low) group has poorer survival. An asterisk with the type name indicates a significant relation to poor prognosis. **b-d** Hazard ratios for rank ordered CNH groups (**b**), rank ordered aneuploidy score groups (**c**) and genome doubling (**d**) in a pan-cancer multivariate Cox proportional-hazards model including: CNH, aneuploidy score, genome doubling, type, age, gender, stage, MSI status and mutations in *TP53*, *MYC*, *KRAS*, *BRAF*, *PI3CK*, *VHL*, *APC* and *PTEN*. Significance is indicated by diamonds. Error bars represent 95% confidence intervals of hazard ratios as calculated by the 2-sided Wald test. Source data are provided as a Source Data file.

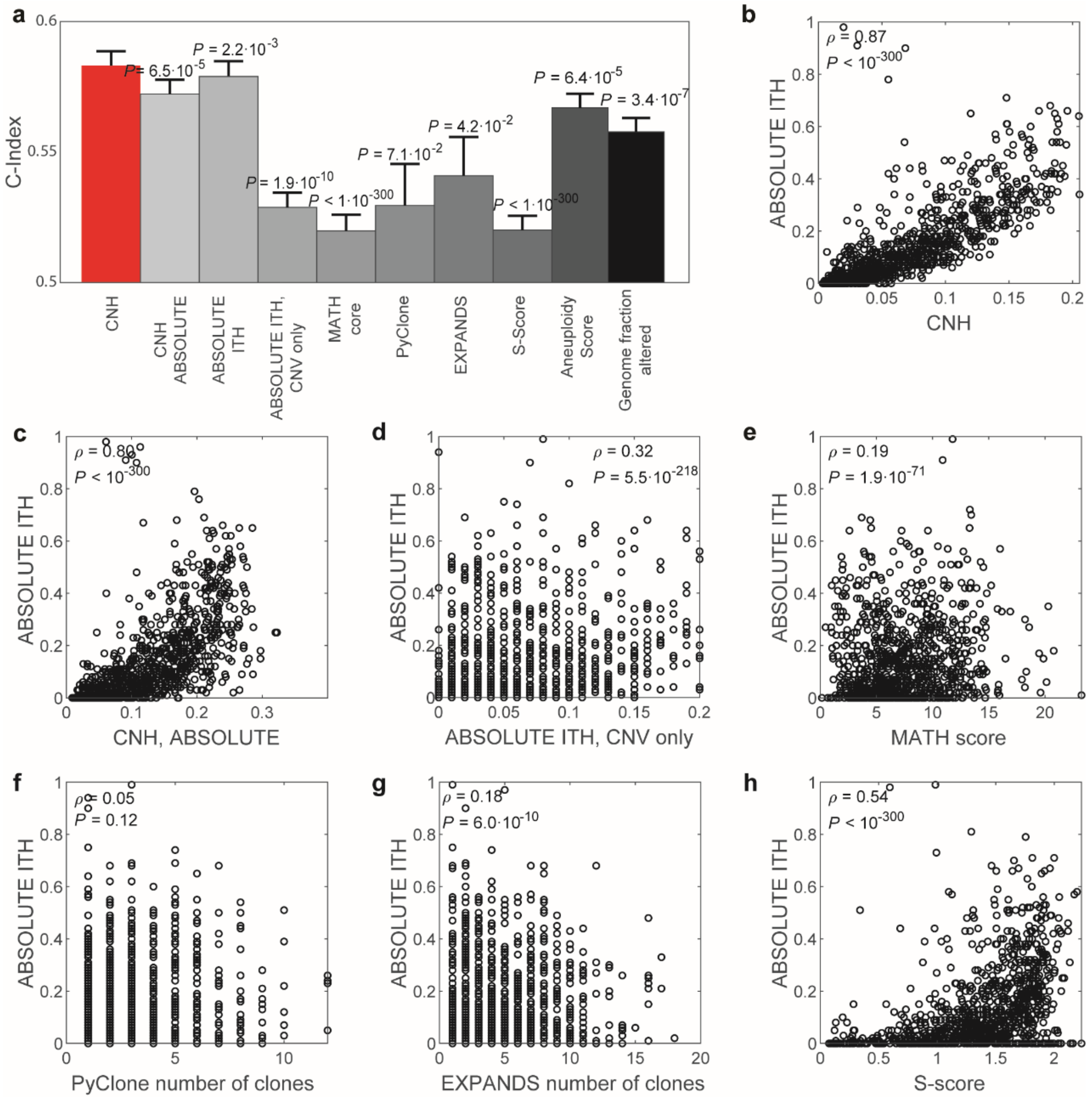

Supplementary Fig. 6 CNH outperforms other measures of ITH in predicting survival rates. a The concordance-index as calculated by the univariable cox hazard model for predicting overall survival in TCGA across cancers is significantly higher for copy number heterogeneity (CNH) than for ABSOLUTE ITH determined from copy number and mutation data (n=9,171 samples), CNH determined with ploidy and purity from ABSOLUTE (n=9,872 samples), ABSOLUTE ITH determined from copy number data only (n=1,255 samples from SARC, LAML, UCEC and LGG), MATH (n=9,030 samples), PyClone (n=1,072 samples), EXPANDS (n=1,129 samples), S-score (n=10,052 samples), Aneuploidy score (n=9,753 samples) and the genome fraction altered (n=10208 samples)[9-12,19,38]. Concordance indices were compared using "CompareC"[46] with the 2-sided test. Error bars indicate standard deviations. **b-h** Spearman's rank correlation to ITH determined by ABSOLUTE using copy number and mutation data for: CNH (**b**), CNH calculated using ploidy and purity determined by ABSOLUTE (**c**), Absolute ITH, calculated using only copy number data (**d**) MATH (**e**), PyClone (**f**), EXPANDS (**g**) and S-score (**h**). CNH, using only copy number data, correlates strongly and best of all methods to ITH determined by ABSOLUTE using copy number and mutation data. Source data are provided as a Source Data file.

	PFI		OS	
	**HR**	***P*-value**	**HR**	***P*-value**
**CNH groups (ref:0%-20%)**				
20%-40%	1.15	1.73E-01	1.21	7.35E-02
40%-60%	1.23	4.42E-02	1.40	2.52E-03
60%-80%	1.32	1.25E-02	1.53	2.58E-04
80%-100%	1.33	1.31E-02	1.51	5.54E-04
**Aneuploidy score groups (ref:0%-20%)**				
20%-40%	1.23	4.40E-02	1.03	7.70E-01
40%-60%	1.25	3.40E-02	1.11	3.51E-01
60%-80%	1.18	1.40E-01	1.05	7.01E-01
80%-100%	1.00	9.76E-01	0.97	8.28E-01
**Stage (ref:1)**				
2	1.61	1.10E-11	1.53	1.58E-08
3	2.46	6.98E-40	2.54	1.12E-37
4	4.75	7.86E-87	4.88	1.21E-81
Age (ref: < **60)**				
60-69	1.10	7.16E-02	1.23	3.75E-04
70-79	1.20	1.74E-03	1.75	1.38E-20
80+	1.27	1.16E-02	2.55	7.69E-28
**Gender (ref:female)**				
Male	1.03	5.61E-01	0.96	4.16E-01
**MSI status (ref:not-MSI)**				
MSI positive	0.99	9.32E-01	0.98	8.40E-01
**Mutation (ref:wild-type)**				
TP53	1.14	1.44E-02	1.15	9.72E-03
KRAS	1.10	2.74E-01	1.13	1.77E-01
BRAF	1.10	4.70E-01	1.06	7.00E-01
MYC	0.73	1.72E-01	0.58	2.95E-02
PTEN	1.13	2.57E-01	1.11	3.30E-01
PIK3CA	0.91	3.82E-01	1.02	8.75E-01
VHL	0.94	7.57E-01	1.02	9.09E-01
APC	0.87	1.74E-01	1.03	7.50E-01
**Genome doubling**				
1 genome doubling	1.15	5.14E-02	1.00	9.82E-01
2 genome doublings	1.16	2.10E-01	1.19	1.24E-01
**Cancer type (ref:ACC)**				
BLCA	0.59	2.59E-03	0.93	7.18E-01
BRCA	0.21	5.53E-18	0.39	5.06E-06
CESC	0.57	4.12E-03	1.09	6.95E-01
CHOL	2.06	7.86E-03	2.18	9.57E-03
COAD	0.52	8.94E-04	0.66	6.84E-02
DLBC	0.31	4.16E-03	0.32	3.50E-02
ESCA	1.23	2.86E-01	2.16	6.81E-04
HNSC	0.36	6.07E-09	0.76	1.75E-01
KICH	0.24	2.12E-05	0.33	3.31E-03
KIRC	0.52	8.46E-04	0.78	2.77E-01
KIRP	0.52	2.22E-03	0.65	9.78E-02
LIHC	1.67	3.39E-03	1.81	4.46E-03
LUAD	0.95	7.56E-01	1.40	9.97E-02
LUSC	0.61	6.76E-03	1.56	2.79E-02
MESO	1.57	3.02E-02	3.55	1.83E-08
OV	1.02	9.17E-01	1.05	8.23E-01
PAAD	1.93	7.73E-04	3.48	2.66E-08
READ	0.38	2.17E-04	0.48	1.49E-02
SKCM	0.98	9.22E-01	1.63	7.44E-02
STAD	0.74	9.99E-02	1.75	7.12E-03
TGCT	0.48	2.05E-03	0.09	9.34E-06
THCA	0.24	2.55E-09	0.13	9.30E-09
THYM	0.33	7.06E-05	0.27	6.35E-04
UCEC	0.46	6.38E-05	0.52	4.55E-03
UCS	1.40	1.82E-01	1.73	5.09E-02
UVM	0.77	2.87E-01	1.23	4.56E-01

Supplementary Table 1 CNH is prognostic for PFI and OS in multivariate analysis. Hazard-ratios (HR) and *P*-values from multivariate Cox proportional-hazards models for

progression free interval (PFI) and overall survival (OS) of 7,081 patients from TCGA as determined by the 2-sided Wald test. Source data are provided as a Source Data file.
