# Supplementary material for: Chromosomal copy number heterogeneity predicts survival rates across cancers
Source: Nat Commun. 2021 May 27;12:3188. doi: 10.1038/s41467-021-23384-6 (PMC8160133; doi:10.1038/s41467-021-23384-6)
Supplement: Supplementary file 3 — Description of Additional Supplementary Files [file 41467_2021_23384_MOESM3_ESM.pdf]

Description of Additional Supplementary Files

File Name: Supplementary Data 1
Description: Supplementary data 1 contains CNH for all patients in the TCGA with primary cancers for which copy number data was available. Further reported values are: CNH without noise filtering, noise in CNH, cancer type, aneuploidy score, survival data, age, gender, tumor stage and grade, MSI status and mutational status of TP53, KRAS, BRAF, MYC, PTEN, PIK3CA, VHL and APC.

File Name: Supplementary Data 2
Description: Supplementary data 2 contains the rank correlation of gene expression to CNH. The correlation to CNH is calculated for all genes, from all primary cancers from which expression data and CNV data was available in the TCGA. In addition the P-value and an indicator whether the gene was used in the gene network of figure 2b are reported.
