# Supplementary material for: Chromosomal copy number heterogeneity predicts survival rates across cancers
Source: Nat Commun. 2021 May 27;12:3188. doi: 10.1038/s41467-021-23384-6 (PMC8160133; doi:10.1038/s41467-021-23384-6)
Supplement: Supplementary file 6 — Reporting Summary [file 41467_2021_23384_MOESM6_ESM.pdf]

Corresponding author(s): Louis Vermeulen
Daniël Miedema

Last updated by author(s): March 23, 2021

Reporting Summary

Nature Research wishes to improve the reproducibility of the work that we publish. This form provides structure for consistency and transparency in reporting. For further information on Nature Research policies, see our Editorial Policies and the Editorial Policy Checklist.

Statistics

For all statistical analyses, confirm that the following items are present in the figure legend, table legend, main text, or Methods section.

n/a	Confirmed	
☐	☒	The exact sample size (*n*) for each experimental group/condition, given as a discrete number and unit of measurement
☐	☒	A statement on whether measurements were taken from distinct samples or whether the same sample was measured repeatedly
☐	☒	The statistical test(s) used AND whether they are one- or two-sided<br>*Only common tests should be described solely by name; describe more complex techniques in the Methods section.*
☐	☒	A description of all covariates tested
☐	☒	A description of any assumptions or corrections, such as tests of normality and adjustment for multiple comparisons
☐	☒	A full description of the statistical parameters including central tendency (e.g. means) or other basic estimates (e.g. regression coefficient) AND variation (e.g. standard deviation) or associated estimates of uncertainty (e.g. confidence intervals)
☐	☒	For null hypothesis testing, the test statistic (e.g. *F*, *t*, *r*) with confidence intervals, effect sizes, degrees of freedom and *P* value noted<br>*Give P values as exact values whenever suitable.*
☒	☐	For Bayesian analysis, information on the choice of priors and Markov chain Monte Carlo settings
☒	☐	For hierarchical and complex designs, identification of the appropriate level for tests and full reporting of outcomes
☐	☒	Estimates of effect sizes (e.g. Cohen's *d*, Pearson's *r*), indicating how they were calculated

Our web collection on statistics for biologists contains articles on many of the points above.

Software and code

Policy information about availability of computer code

Data collection	The score-client was used to download data for the ICGC dataset. No other software was used to collect data
Data analysis	HapSeg (v1.1.1) and ABSOLUTE (v1.0.6) were used to analyze SNParray data. BWA (v0.7.12) was used for read alignment of single-cell data and R-package Aneufinder (v1.14) was used to further analyze single-cell data. Cox proportional-hazards models were constructed with the "coxph" function from the "survival" package (v 3.2.7) and Concordance-indices were determined and compared using "CompareC" function from the "CompareC" package (v1.3.1). R-version used was R-3.6.3.<br><br>All other analyses were performed in, and all code was written in MATLAB R2019a.

For manuscripts utilizing custom algorithms or software that are central to the research but not yet described in published literature, software must be made available to editors and reviewers. We strongly encourage code deposition in a community repository (e.g. GitHub). See the Nature Research guidelines for submitting code & software for further information.

Data

Policy information about availability of data

All manuscripts must include a data availability statement. This statement should provide the following information, where applicable:

- Accession codes, unique identifiers, or web links for publicly available datasets
- A list of figures that have associated raw data
- A description of any restrictions on data availability

TCGA data analysed in this manuscript can be downloaded from http:///gdac.broadinstitute.org. The BriTROC-1 data is available on: https://bitbucket.org/britroc/cnsignatures/src/master/ and raw data can be obtained by contacting James Brenton, corresponding author of the original publication: James.Brenton@cruk.cam.ac.uk. CAIRO2 data measured with arrayCGH is publicly available from gene expression omnibus (GEO), accession code GSE36864 https://

www.ncbi.nlm.nih.gov/geo/query/acc.cgi?acc=GSE36864, and measured with shallow-whole genome sequencing is available from the European Genome Archive (EGA), accession code EGAS00001002617, https://ega-archive.org/studies/EGAS00001002617. Data from TRACERX used in this study is available from Jamal-Hanjani et al (ref). Data from ICGC is under restricted access, available from https://dcc.icgc.org/repositories. Single cell karyotype of CRC samples and SNP data measured from bulk of the oesophageal and ovarian organoids is available from the EGA, accession code EGAS00001004702, https://ega-archive.org/studies/EGAS00001004702.
Source data are available as a Source Data file. The remaining data are available within the Article, Supplementary Information or available from the authors upon request.

Field-specific reporting

Please select the one below that is the best fit for your research. If you are not sure, read the appropriate sections before making your selection.

☒ Life sciences ☐ Behavioural & social sciences ☐ Ecological, evolutionary & environmental sciences

For a reference copy of the document with all sections, see nature.com/documents/nr-reporting-summary-flat.pdf

Life sciences study design

All studies must disclose on these points even when the disclosure is negative.

Sample size	In all analyses of TCGA, only samples from a primary tumour or a primary blood derived cancer were included (n = 10,578 with copy number data; n = 10,208 after noise filtering). For each analysis, all available data dat passed QC of primary cancers from all 33 cohorts in TCGA were included. For data from the BriTROC-1 cohort all 253 primary and relapse tumour samples from all 132 patients were included. For data from the TRACERx study,consisting of 303 samples from 100 patients, all patients with more than one sample were analyzed. For the CAIRO2 data, all 96 samples on which copy number data from both arrayCGH and NGS measurements were available were included.
Data exclusions	Single-cell karytotype sequencing data failing QC from Aneufinder (v1.14) were excluded. From TCGA data, metastatic and non-primary cancers were excluded from the analysis. QC was developed and applied to copy number data to exclude noisy segments and samples. Further, no data was excluded.
Replication	The survival analysis were performed with identical criteria (rank order on copy number heterogeneity, split in two groups) for each of the 33 cancer types in TCGA. Hence, no replication of the same tissue type were done, but the survival were repeated 33 times in cohorts from different tissues. The pan-cancer analysis was replicated using an independent data set of 1,326 samples with copy number and survival data from 19 studies from ICGC that are not also part of TCGA. All attempts at replication were succesfull.<br>The experiments involving live cell imaging of organoids were repeated 3 times for each organoid line.
Randomization	Not relevant to our study: our analyses were retrospective.
Blinding	Not relevant to our study: our analyses were retrospective.

Reporting for specific materials, systems and methods

We require information from authors about some types of materials, experimental systems and methods used in many studies. Here, indicate whether each material, system or method listed is relevant to your study. If you are not sure if a list item applies to your research, read the appropriate section before selecting a response.

Materials & experimental systems

n/a	Involved in the study	
☒	☐	Antibodies
☒	☐	Eukaryotic cell lines
☒	☐	Palaeontology and archaeology
☒	☐	Animals and other organisms
☐	☒	Human research participants
☒	☐	Clinical data
☒	☐	Dual use research of concern

Methods

n/a	Involved in the study	
☒	☐	ChIP-seq
☒	☐	Flow cytometry
☒	☐	MRI-based neuroimaging

Human research participants

Policy information about studies involving human research participants

Population characteristics	Tumor material was collected in this study for single-cell karyotype sequencing from colorectal cancer patients with peritoneal metastasis. 7 samples (4 from primary tumors, 3 from peritoneal metastases) from 4 patients (3 male, 1 female) were analyzed.
Recruitment	No patients were specifically recruited for this study: we used available material.
Ethics oversight	Patient samples were collected according to Dutch research guidelines, as described in "Human Tissue and Medical Research: Code of conduct for Responsible use". The procedure was overseen by the Medical ethical review committee at the VU Medical Center.

Note that full information on the approval of the study protocol must also be provided in the manuscript.
